# Eligibility criteria in clinical trials in breast cancer: a cohort study

**DOI:** 10.1186/s12916-023-02947-y

**Published:** 2023-07-03

**Authors:** Katarzyna Szlezinger, Katarzyna Pogoda, Agnieszka Jagiełło-Gruszfeld, Danuta Kłosowska, Andrzej Górski, Jan Borysowski

**Affiliations:** 1 Pharmacovigilance Department, Office for Registration of Medicinal Products, Medical Devices and Biocidal Products, Aleje Jerozolimskie 181C, 02-222 Warsaw, Poland; 2 Department of Breast Cancer and Reconstruction Surgery, Maria Sklodowska-Curie National Research Institute of Oncology, Roentgena 5, 02-781 Warsaw, Poland; 3 Department of Clinical Immunology, Medical University of Warsaw, Nowogrodzka 59, 02-006 Warsaw, Poland; 4 Bacteriophage Laboratory, Department of Phage Therapy, Hirszfeld Institute of Immunology and Experimental Therapy, Polish Academy of Sciences, Rudolfe Weigla 12, 53-114 Wrocław, Poland

**Keywords:** Breast cancer, Clinical trial, Eligibility criteria, Enrollment criteria, Exclusion criteria, Elderly, Older adult, Comorbidity, Performance status

## Abstract

**Background:**

Breast cancer (BC) is the most common cancer type in women. The purpose of this study was to assess the eligibility criteria in recent clinical trials in BC, especially those that can limit the enrollment of older patients as well as those with comorbidities and poor performance status.

**Methods:**

Data on clinical trials in BC were extracted from ClinicalTrials.gov. Co-primary outcomes were proportions of trials with different types of the eligibility criteria. Associations between trial characteristics and the presence of certain types of these criteria (binary variable) were determined with univariate and multivariate logistic regression.

**Results:**

Our analysis included 522 trials of systemic anticancer treatments started between 2020 and 2022. Upper age limits, strict exclusion criteria pertaining to comorbidities, and those referring to inadequate performance status of the patient were used in 204 (39%), 404 (77%), and 360 (69%) trials, respectively. Overall, 493 trials (94%) had at least one of these criteria. The odds of the presence of each type of the exclusion criteria were significantly associated with investigational site location and trial phase. We also showed that the odds of the upper age limits and the exclusion criteria involving the performance status were significantly higher in the cohort of recent trials compared with cohort of 309 trials started between 2010 and 2012 (39% *vs* 19% and 69% *vs* 46%, respectively; *p* < 0.001 for univariate and multivariate analysis in both comparisons). The proportion of trials with strict exclusion criteria was comparable between the two cohorts (*p* > 0.05). Only three of recent trials (1%) enrolled solely patients aged 65 or 70 and older.

**Conclusions:**

Many recent clinical trials in BC exclude large groups of patients, especially older adults, individuals with different comorbidities, and those with poor performance status. Careful modification of some of the eligibility criteria in these trials should be considered to allow investigators to assess the benefits and harms of investigational treatments in participants with characteristics typically encountered in clinical practice.

**Supplementary Information:**

The online version contains supplementary material available at 10.1186/s12916-023-02947-y.

## Background

Breast cancer (BC) is the most common cancer type and the second most common cause of cancer-related deaths in women [1]. According to the World Health Organization (WHO) statistics, in 2020, 2.3 million women were diagnosed with BC and 685,000 women died of BC [2]. As of the end of 2020, there were 7.8 million women alive who were diagnosed with BC in the past 5 years [2]. The American Cancer Society (ACS) estimates that over 280,000 of new cases of invasive BC will be diagnosed in the USA in 2022 and more than 43,000 women will die of BC [3].

Risk of BC increases with age. The median age at the time of diagnosis is 62 [3]. Almost one third of new cases of BC are diagnosed in women aged 70 and older [4]. Although the incidence of BC in women aged 80 and older is slightly less, the mortality rate in patients from this age group is actually higher [5]. However, in spite of high prevalence of cancer in older patients, their involvement in oncology clinical trials has been traditionally inadequate [6, 7]. This results in limited evidence base for the treatment of older adults with BC and hence undertreatment of patients from higher age groups [8, 9].

It is also known that many patients with BC have comorbidities. For instance, according to the US statistics, up to 42% of women with BC have at least one comorbidity at the time of diagnosis [10]. The most common comorbidities occuring in patients with BC include among others hypertension, ischemic heart disease, heart failure, and depression [10, 11]. Apart from specific comorbidities, an important aspect in the assessment of a patient with cancer is also the performance status—a score that reflects the patient’s capability to perform different activities of daily living without the help of others [12].

Over the last decade, considerable efforts have been made by regulatory agencies, cancer societies, and advocacy organizations to improve the enrollment of older patients, as well as those with comorbidities and poor performance status, in clinical trials of anticancer treatments [7]. For instance, broadening of the overly strict eligibility criteria used in cancer clinical trials was postulated by the American Society of Clinical Oncology (ASCO) and Friends of Cancer Research Joint Research Statement published in 2017 [13]. The enrollment of older adults in cancer clinical trials is also promoted by the Food and Drug Administration (FDA) which developed relevant guidance document for the pharmaceutical industry [14].

One of the key barriers to the enrollment of patients in cancer clinical trials is the eligibility criteria including upper age limits, the criteria requiring adequate performance status of the patient, and those involving certain comorbidities [13, 15–17]. The purpose of this study was to assess the eligibility criteria in recent clinical trials in BC, especially those that can limit the enrollment of older adults as well as individuals with comorbidities and poor performance status. To our knowledge, our study is the first to provide comprehensive analysis of these criteria.

## Methods

### Selection of eligible clinical trials

Our study included clinical trials registered with ClinicalTrials.gov (CT.gov; https://www.clinicaltrials.gov/). CT.gov is a publically available Web-based resource that provides access to information on clinical studies concerning a wide range of diseases and conditions. It is maintained by the National Library of Medicine (NLM) at the National Institutes of Health (NIH) and enables the responsible parties to register trials being conducted all over the world. CT.gov is the largest register of clinical studies; over the last decade, it emerged as a very important source of information on clinical trials [18]. For instance, a number of studies on the eligibility criteria used in clinical trials were performed based on the data contained in this register [16, 17, 19].

Our analysis focused on recent clinical trials of systemic anticancer treatments in BC. These were searched for in CT.gov on July 12, 2022 using the search term “breast cancer” (field “Condition or disease”). Taking advantage of Advanced search function, we selected for analysis interventional studies with the primary purpose “Treatment” that were started on 01/01/2020 or later (registered trials with status “Not yet recruiting” and the planned start date by the end of 2022 were also included). We focused on phase 1, 2, 3, and 4 trials which are most important for the collection of data on the efficacy and safety of investigational drugs. Early phase 1 trials, trials with phase classified as “Not applicable” (this category generally includes studies of different therapeutic procedures and medical devices) as well as those with the recruitment status “Suspended,” “Withdrawn,” and “Unknown” were excluded. We also excluded trials performed on healthy volunteers (anticancer drugs are generally tested on patients with cancer and it is these trials that allow the investigators to collect the data that are most important). Since we were interested in systemic treatments which constitute the mainstay of anticancer therapy, we excluded trials of pharmacological interventions applied locally and non-pharmacological interventions. Trials enrolling patients with multiple cancer types were also excluded. Record of each of the potentially eligible trials was manually reviewed to ensure that it meets all the inclusion criteria.

### Data extraction and analysis

Using a functionality of CT.gov from record of each eligible trial, we imported into Excel a range of data including title, condition/disease, CT.gov identifier, status, trial registration date and start date, sample size, intervention name and type, trial phase, center location, and the sponsor. The sponsors were divided into three categories: (1) Industrial (trials in which the pharmaceutical industry was either the primary sponsor or a “collaborator” according to the terminology used by CT.gov); (2) NIH (trials in which the NIH was either the primary sponsor or a collaborator); (3) Other.

Timeframe for the primary endpoint assessment and data on the eligibility criteria were extracted manually from relevant sections in records of individual trials deposited in CT.gov. In analysis of the upper age limits, in line with the limits of main age categories adopted by CT.gov [https://www.clinicaltrials.gov/ct2/search/advanced], we considered individuals aged 65 and older as older adults. The assessment of the eligibility criteria concerning comorbidities was based on the classification system developed by Lewis et al. with a view to investigating the exclusion of older patients from clinical trials in oncology [20]; it was also used in other studies on the exclusion of older adults from clinical trials including a study from our group [16, 21]. The classification includes a comprehensive set of comorbidities of different organs and systems including the liver, the kidneys, bone marrow, the cardiovascular system, and the pulmonary system. Moreover, it includes psychiatric disorders and prior or concurrent malignancies. Overall, the classification dinstinguishes two main categories of the exclusion criteria: moderate and strict. While the former allow for mild abnormalities, strict criteria require normal or almost normal system/organ function and/or laboratory parameters. If a trial had both strict and moderate criteria related to the same organ/system, it was classified as having strict criteria. The details of the classification are available at (https://theoncologist.onlinelibrary.wiley.com/doi/suppl/10.1634/theoncologist.2014-0093).

We also recorded the exclusion criteria involving inadequate performance status of the patient. These were mostly based on the Eastern Cooperative Oncology Group (ECOG) scale. In rare cases of the criteria referring to Karnofsky scale, we converted relevant grades into the equivalent values in the ECOG scale as reported elsewhere [16].

All the data were inserted into an Excel spreadsheet, and analyzed in Excel. Data extraction and analysis were performed independently by two investigators (K.S. and J.B.). Discrepancies were resolved through consensus, and in cases when consensus could not be reached, the third investigator (K.P.) was consulted.

Co-primary outcomes of this study included proportion of trials with an upper age limit, at least one strict exclusion criterion concerning a comorbidity, and those excluding patients with inadequate performance status (ECOG > 1).

### Statistical analysis

Descriptive statistics were used to analyze included trials. Discrete variables were presented as absolute numbers and percentages, and continuous variables as medians with interquartile ranges. In order to determine the associations between certain trial characteristics and the odds of the exclusion of older adults as well as patients with poor performance status and comorbidities (binary variables), univariate and multivariate logistic regression was used. Each of the covariates was included in both univariate and multivariate model. The results of logistic regression were presented as odds ratios (ORs) and 95% confidence intervals (CIs). Significance of individual covariates used in logistic regression models was determined with Wald test. Temporal trends in the proportion of different types of the exclusion criteria were determined with univariate logistic regression. Chi-square test was used to compare the frequency of different types of the eligibility criteria between the cohort of recent trials and the historical cohort. Statistical calculations were performed using R package. The *P* value level of significance was specified at 0.05.

## Results

### Characteristics of included trials

**Fig. 1:**
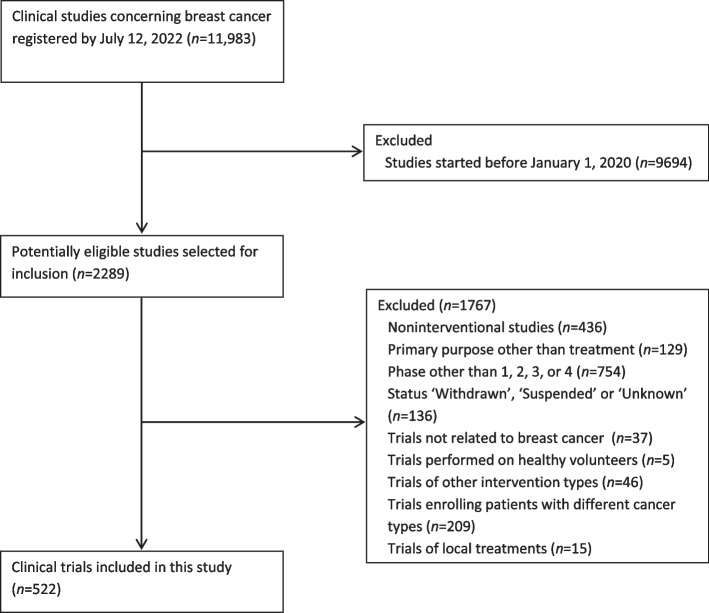
Selection of clinical trials in breast cancer started between 2020 and 2022

Trial selection is shown in Fig. 1. The initial search yielded 11,983 trials, of which 522 met the inclusion criteria. Included trials are listed in Additional file 1.

Detailed characteristics of included trials are shown in Table 1. Most of the trials were phase 2 trials (*n* = 284; 54%), followed by phase 3 trials (*n* = 96; 18%). In total, 337 (65%) trials enrolled patients with advanced BC. Many trials involved a combination of hormonal therapy and targeted therapy (*n* = 109; 21%), chemotherapy and targeted therapy (*n* = 88; 17%), and targeted therapy (*n* = 82; 16%). The median number of participants was 75 (interquartile range (IR), 40–210). Most of the trials were sponsored by the pharmaceutical industry (*n* = 285; 55%). The median timeframe for primary endpoint assessment was 80 weeks (IR range, 24–144). The majority of trials were performed at centers located in Asia (*n* = 186; 36%), North America (*n* = 134; 26%) or Europe (*n* = 129; 25%).

**Table 1:** Characteristics of clinical trials in breast cancer started between 2020 and 2022. Shown are the numbers and proportions of trials with certain characteristics

	*n*	%
**Intervention type**		
C	37	7
C + T	88	17
T	82	16
H	34	7
H + T	109	21
I	23	4
I + C	39	7
O	110	21
**Randomization**		
No	316	61
Yes	206	39
**Phase**		
1	65	12
1/2	44	8
2	284	54
2/3	13	2
3	96	18
4	20	4
**Breast cancer**		
Early	185	35
Advanced	337	65
**Breast cancer type**		
HER2 +	144	28
HR + HER2 −	173	33
TNBC	116	22
Multiple^a^	89	17
**Sponsor**		
Pharmaceutical industry^b^	285	55
NIH	21	4
Other	216	41
**Center location**		
Asia	186	36
North America	134	26
Europe	129	25
Other	12	2
Intercontinental	61	12
**Multicenter trials**		
No	304	58
Yes	218	42
**International trials**		
No	444	85
Yes	78	15

*Abbreviations*: *C* Chemotherapy, *H* Hormonal therapy, *I* Immunotherapy, *NIH* National Institutes of Health, *O* Other, *T* Targeted therapy, *TNBC* Triple-negative breast cancer

^a^Trials enrolling patients with more than one breast cancer type

^b^Any involvement of the pharmaceutical industry

Percentages may not always total 100% due to rounding error

### Age limits

Detailed data on the age limits used in included clinical trials are shown in Table 2.

**Table 2:** Exclusion criteria in clinical trials in breast cancer started between 2020 and 2022. Shown are the numbers and proportions of trials excluding patients based on age, comorbidities, and the performance status. A trial was considered as excluding patients based on age if it had any upper age limit, based on comorbidities if it had at least one strict exclusion criterion, and based on the performance status if it excluded patients with Eastern Cooperative Oncology Group (ECOG) grade > 1. Some trials may have had more than one type of the exclusion criteria. The assessment of the exclusion criteria concerning comorbidities is based on the classification system developed by Lewis et al. [20]

	*n*	%
**Upper age limits (years)**		
≤ 65	13	2
66–70	61	12
71–75	86	16
76–80	11	2
81–85	1	0
> 85	32	6
No limit	318	61
**Comorbidities**		
Malignancy		
Strict	46	9
Moderate	309	59
No restriction	167	32
Hepatic		
Strict	265	51
Moderate	182	35
No restriction	75	14
Cardiac		
Strict	246	47
Moderate	176	34
No restriction	100	19
Other cardiovascular		
Strict	95	18
Moderate	89	17
No restriction	338	65
Renal		
Strict	217	42
Moderate	212	41
No restriction	93	18
Pulmonary		
Strict	112	21
Moderate	110	21
No restriction	300	57
Psychiatric		
Strict	47	9
Moderate	155	30
No restriction	320	61
Bone marrow		
Strict	9	2
Moderate	432	83
No restriction	81	16
**Allowable ECOG grade**		
0–1	360	69
0–2	112	21
0–3	3	1
Any	47	9

*Abbreviations*: *ECOG* Eastern Cooperative Oncology Group

Percentages may not always total 100% due to rounding error

Overall, 204 trials (39%) had an upper age limit. The limits mostly fell within the range between 71 and 75 years of age (*n* = 86; 16%). Only three trials (1%) enrolled solely participants aged 65 or 70 and older (NCT04305834; NCT04272801; NCT04134598; none of these had an upper age limit). In addition, two other trials enrolled participants aged 50 (NCT04852887) or 51 (NCT05297617) and older, but the latter trial had an upper age limit (70).

Table 3 presents proportions of trials with the age limits stratified by main trial characteristics. Of note, high proportion of trials excluding patients based on age was noted not only in early phase, but also in late phase trials. For instance, proportion of trials with an upper age limit was almost three times higher among phase 3 trials compared with phase 1 trials (46% and 17%, respectively; Table 3).

**Table 3:** Exclusion of patients from clinical trials in breast cancer started between 2020 and 2022 stratified by main trial characteristics. Shown are the numbers and proportions of trials with certain characteristics excluding patients based on age, comorbidities, and poor performance status. A trial was considered as excluding patients based on age if it had any upper age limit, based on comorbidities if it had at least one strict exclusion criterion, and based on the performance status if it excluded patients with Eastern Cooperative Oncology Group (ECOG) grade > 1. The assessment of the exclusion criteria concerning comorbidities is based on the classification system developed by Lewis et al. [20]

Trial characteristic	Total number of trials	Exclusion based on age limits		Exclusion based on comorbidities		Exclusion based on performance status		Exclusion overall^a^	
	***n***	***n***	**%**	***n***	**%**	***n***	**%**	***n***	**%**
**Phase**									
1	65	11	17	41	63	45	69	61	94
1/2	44	17	39	35	80	26	59	42	95
2	284	115	40	240	85	201	71	272	96
2/3	13	10	77	11	85	9	69	13	100
3	96	44	46	65	68	66	69	90	94
4	20	7	35	12	60	13	65	15	75
**Continent**									
North America	134	15	11	107	80	72	54	125	93
Europe	129	46	36	108	84	90	70	121	94
Asia	186	127	68	147	79	146	78	182	98
Other	12	5	42	7	58	5	42	9	75
Intercontinental	61	11	18	35	57	47	77	56	92
**Intervention type**									
C	37	23	62	27	73	21	57	33	89
C + T	88	48	55	78	89	72	82	87	99
T	82	29	35	65	79	58	71	76	93
H	34	14	41	22	65	17	50	31	91
H + T	109	27	25	76	70	77	71	100	92
I	23	4	17	20	87	15	65	22	96
I + T	39	18	46	28	72	30	77	38	97
O	110	41	37	88	80	70	64	106	96
**Breast cancer**									
Advanced	185	83	45	151	82	134	72	175	95
Early	337	121	36	253	75	226	67	318	94
**Sponsor**									
Pharmaceutical industry	285	81	28	215	75	217	76	273	96
NIH	21	2	10	16	76	8	38	18	86
Other	216	121	56	173	80	135	63	202	94

*Abbreviations*: *C* Chemotherapy, *H* Hormonal therapy, *I* Immunotherapy, *NIH* National Institutes of Health, *O* Other, *T* Targeted therapy

^a^Trials excluding patients based on age, comorbidities, or the performance status. Some trials may have had more than one type of the exclusion criteria

Using logistic regression, we identified the factors affecting the odds of a trial having an upper age limit. A range of basic trial characteristics likely to influence the presence of the age limits were included as covariates to logistic regression models. Detailed results of logistic regression analyses are presented in Table S1 (Additional file 2). Multivariate analysis showed that the covariates with the most clear association with the risk of the presence of an upper age limit included center location and trial phase. In particular, the odds of a trial excluding older patients based on an upper age limit were higher in trials performed in Asia (adjusted odds ratio (aOR), 18.49; confidence interval (CI), 9.13–40.05; *p* < 0.001) and Europe (aOR, 4.16; CI, 2.02–9.0; *p* < 0.001) relative to trials performed in the USA, phase 3 (aOR, 4.72; CI, 1.8–13.13; *p* = 0.002), phase 1/2 (aOR, 4.35; CI, 1.48–13.35; *p* = 0.008), and phase 2/3 trials (aOR, 12.35; CI, 2.34–82.26; *p* = 0.004) relative to phase 1 trials as well as in trials with sponsors from category “Other” (aOR, 2.07; CI, 1.25–3.43; *p* = 0.005) relative to trials sponsored by the pharmaceutical industry. The odds of an upper age limit were significantly lower in trials involving a combination of hormonal therapy and targeted therapy (aOR, 0.26; CI, 0.09–066; *p* = 0.005).

We also compared the frequency of the upper age limits between the cohort of recent trials and a historical cohort of 309 clinical trials that met the same inclusion criteria as recent trials, but were started between 2010 and 2012 (detailed data on the historical cohort not shown). We found that 58 (19%) of the trials from the historical cohort had upper age limits. The difference in the frequency of the upper age limits between the cohorts was statistically significant (*p* < 0.001; Table 4). This finding was also confirmed by multivariate analysis after adjustment for a number of the covariates related to the trials including breast cancer stage, intervention type, phase, sample size, sponsor, center location, and timeframe for primary endpoint assessment (aOR, 2.22; CI, 1.39–3.6; *p* < 0.001).

**Table 4:** Comparison of the exclusion of patients between cohort of trials started between 2020 and 2022 and historical cohort (trials started between 2010 and 2012). Shown are the numbers and proportions of trials excluding patients based on age, comorbidities, and the performance status. A trial was considered as excluding patients based on age if it had any upper age limit, based on comorbidities if it had at least one strict exclusion criterion, and based on the performance status if it excluded patients with Eastern Cooperative Oncology Group (ECOG) grade > 1. The assessment of the exclusion criteria concerning comorbidities is based on the classification system developed by Lewis et al. [20]. Statistical significance was determined with chi-square test. *P* value level of significance was specified at 0.05

	Trials started between 2020 and 2022	Trials started between 2010 and 2012	*P*
Total number of trials	522	309	-
Trials excluding patients based on age	204 (39%)	58 (19%)	< 0.001
Trials excluding patients based on comorbidities	404 (77%)	223 (72%)	0.19
Trials excluding patients based on the performance status	360 (69%)	142 (46%)	< 0.001

### Exclusion criteria concerning comorbidities

Detailed data on the exclusion criteria concerning comorbidities are presented in Table 2. Overall, 404 trials (77%) had at least one strict exclusion criterion involving a comorbidity. These included mostly liver function disorders (*n* = 265; 51%), followed by cardiac diseases (*n* = 246; 47%) and renal function disorders (*n* = 217; 42%). While many trials listed the exclusion criteria related to bone marrow function (*n* = 441; 84%) and malignancies (*n* = 355; 68%; Table 2), most of these were moderate according to classification by Lewis et al. [20]. Proportion of trials with strict exclusion criteria concerning comorbidities stratified by main trial characteristics is shown in Table 3. As was the case with the upper age limits, the criteria concerning comorbidities were also common in late phase trials (e.g., 68% of phase 3 trials had at least one strict exclusion criterion concerning comorbidity). We also found that 39 out of 204 (19%) trials with an upper age limit did not have any strict exclusion criterion concerning comorbidity.

On multivariate analysis, the odds of a trial having at least one strict exclusion criterion concerning comorbidity were higher in phase 2 trials relative to phase 1 trials (aOR, 2.76; CI, 1.4–5.39; *p* = 0.002) and lower in trials performed at centers located in continents other than North America, Europe, and Asia (aOR, 0.26; CI, 0.06–1.04; *p* = 0.04) as well as in intercontinental trials (aOR, 0.42; CI, 0.19–0.94; *p* = 0.03) relative to trials with centers located in North America. Detailed results of this analysis are shown in Table S2 (Additional file 3).

In the historical cohort (trials started between 2010 and 2012), strict exclusion criteria concerning comorbidities were listed in 226 (73%) trials. The difference between this cohort and the cohort of recent trials was not statistically significant (*p* = 0.19; Table 4). Multivariate analysis also did not show that either cohort is associated with higher odds of the presence of strict exclusion criteria involving comorbidities (*p* = 0.38).

### Exclusion based on the performance status of the patient

The criteria excluding patients based on inadequate performance status were listed in 475 trials (91%). Most of these excluded individuals with ECOG > 1 (*n* = 360; 69%; co-primary outcome) or ECOG > 2 (*n* = 112; 21%). ECOG 0–3 was allowable in only three (1%) trials. Proportion of trials excluding patients with ECOG > 1 stratified by main trial characteristics is shown in Table 3. This proportion was high in early and late phase trials (for instance in both phase 1 and phase 3 trials it was 69%; Table 3). We also noted that as many as 58 out of 204 (28%) trials with an upper age limit allowed for the inclusion of patients with ECOG > 1.

On multivariate analysis, the odds of a trial excluding patients with ECOG > 1 were higher in trials performed in Asia (aOR, 4.31; CI, 2.39–7.9; *p* < 0.001), Europe (aOR, 2.16; CI, 1.21–3.92; *p* = 0.009), and intercontinental trials (aOR, 3.46; CI, 1.54–8.14; *p* = 0.003) relative to trials with investigational sites located in North America as well as in trials in which a combination of chemotherapy and targeted therapy was assessed relative to trials of chemotherapy (aOR, 2.59; CI, 1.0–6.4; *p* = 0.03). The odds were lower in trials enrolling patients with advanced cancer relative to studies focusing on early cancer (aOR, 0.45; CI, 0.27–0.74; *p* = 0.002) and in trials with sponsors from category “Other” relative to studies sponsored by the pharmaceutical industry (aOR, 0.35; CI, 0.21–0.57; *p* < 0.001; Table S3 available in Additional file 4).

We also found that 142 (46%) trials from the historical cohort excluded patients with ECOG > 1 (Table 4). The difference between this cohort and the cohort of recent trials was statistically significant (*p* < 0.001). This finding was confirmed by multivariate logistic regresssion that showed that the cohort of recent trials was associated with higher odds of the exclusion of patients with ECOG > 1 (aOR, 2.05; CI, 1.41–2.98; *p* < 0.001).

Overall, 493 trials (94%) started between 2020 and 2022 had either an upper age limit, at least one strict exclusion criterion concerning a comorbidity, or a criterion involving inadequate performance status of the patient. In the historical cohort, the number of trials either explicitly or implicitly excluding older adults was 259 (84%).

## Discussion

Our study has shown a considerable scale of the exclusion from recent clinical trials in BC of older patients as well as those with comorbidities and poor performance status. First of all, 39% of the analyzed trials excluded potential participants based on an upper age limit. Remarkably, proportion of trials with upper age limits increased more than twofold over the last 10 years. However, the key factor contributing to this is likely increase in proportion of trials performed in Asia. Center location in an Asian country was the strongest predictor of the odds of the presence of the upper age limits, with proportion of Asian sites increasing from 15% in the historical cohort to 36% in the cohort of recent trials. Most of recent trials with Asian sites (*n* = 161; 31%) were performed in China. This finding reflects rapidly growing scale of oncology clinical research in China [22].

Common use of the upper age limits in Chinese clinical trials may be to some extent associated with epidemiology of BC in this country. The mean age at diagnosis of BC in China is 49–55, which is a value clearly lower compared with Western countries. Furthermore, proportion of older patients with BC in China is lower by approx. 50% compared with the USA [23]. However, it also needs to be underscored that, in general, China has the largest population of older adults in the world [24]. This implies a proportional increase in the number of older patients with BC. Therefore, in our opinion, clinical trials in China should be to some extent more inclusive to older patients. This problem is important because in China, like in Western countries, BC is the most common cancer type and one of the leading causes of cancer-related deaths [23].

In general, the use of upper age limits in clinical trials is problematic due to considerable heterogeneity of aging. This means that in many cases there is no strict correlation between an older adult’s chronological age and his/her biological age [25]. Therefore, the upper age limits used in clinical trials are in principle arbitrary and very rarely justified (for instance, 6 (1%) trials from our sample were dedicated to premenopausal women; in such cases it is justifiable to exclude older patients). However, with the exception of such rare cases, rather than to exclude patients based on an upper age limit, investigators should consider the use of other justified criteria in order to ensure the safety of trial’s participants. Comprehensive geriatric assessment (CGA), especially the identification of frail individuals is recommended to determine actual biological age and guide type of therapy in older patients [26, 27]. Of note, 19% and 28% of the trials with an upper age limit did not have any strict exclusion criterion concerning comorbidity and allowed for the inclusion of patients with ECOG > 1, respectively. Thus these trials, excluding older patients based solely on the age, in fact accepted younger individuals with poorer state of health.

Intuitively, one could assume that the most stringent eligibility criteria are used in early phase trials where the data on the safety of investigational drugs are most limited. Therefore, an unexpected finding was the higher odds of the presence of upper age limits in phase 1/2, 2/3, and 3 trials relative to phase 1 trials. However, in fact these findings are in line with the results of previous studies which showed that upper age limits were less common in phase 1 trials compared with trials of later phases [16, 28].

Another finding from our study that merits particular attention is a very low proportion of trials dedicated exclusively to older patients (1%). In the absence of such trials, the only source of data on the effects of investigational drugs in individuals from higher age groups are subgroup analyses of trials enrolling both younger and older participants [29]. We believe that more trials dedicated to older adults are needed to obtain results ensuring optimal clinical care of patients from this age group.

Apart from the eligibility criteria, there are also other important barriers limiting the enrollment of older adults in cancer clinical trials. These include especially provider, patient, and caregiver factors. For instance, providers may have concerns for toxicity of the investigational drugs or believe that older patients generally should not be enrolled. Patients themselves may have concerns about the efficacy and safety of new treatments [15]. Thus, while the relaxation of some of the eligibility criteria would have resulted in substantial increase in the enrollment of older adults [20, 30], efforts aiming to reduce other barriers are required to ensure adequate representation of patients from higher age groups in cancer clinical trials [7, 15].

We also found that very high proportion of trials (77%) had strict exclusion criteria concerning comorbidities. Furthermore, proportion of trials with these criteria did not significantly decrease over the last 10 years. Exclusion criteria pertaining to comorbidities aim to ensure the homogeneity of the trial’s sample and the safety of participants. However, overly restrictive criteria limit the generalizability of the trial’s results, the accrual of patients, and their access to new drugs [13, 31]. In fact, comorbidities are one of the key factors limiting the enrollment of patients in oncology clinical trials [30, 32].

One of the exclusion criteria that seem to be particularly problematic concern other malignancies. We showed that 9% of the trials excluded patients with a history of malignancy (a strict criterion) and as many as 59% trials excluded patients with a concurrent malignancy (a moderate criterion). Such a great scale of the exclusion of patients with other malignancies from clinical trials in BC does not seem to be in accord with the recommendations contained in the Joint Research Statement by the ASCO and Friends of Cancer Research [13] as well as a guidance document developed by the FDA [33] which aim to promote, among others, the enrollment of patients with prior and concurrent malignancies in oncology clinical trials.

In this context, it needs to be underscored that comorbidities are also common in older patients with BC [34, 35]. Therefore, the use of restrictive eligibility criteria concerning comorbidities substantially reduces the likelihood of the enrollment of older adults in clinical trials in BC.

We also found that substantial proportion (69%) of trials excluded patients with inadequate performance status (ECOG > 1). In general, the performance status is widely used to assess prognosis in cancer patients including treatment toxicity risk prediction [12, 36]. However, it may not be an ideal predictor of safety of contemporary anticancer treatments that are generally less toxic compared with many chemotherapeutics used in the past [36]. Importantly, inclusion in clinical trials of patients with poor performance status is recommended by the ASCO and Friends of Cancer Research [37].

To our knowledge, so far only two studies were performed to assess the eligibility criteria in BC. Generally, they showed a high rate of the exclusion from clinical trials in BC of older patients as well as those with comorbidities and poor performance status [17, 38]. However, these studies had some limitations (e.g., they focused on trials of selected phases [17, 38] or those sponsored by certain institutions [17], or presented pooled results of analyses of trials concerning BC and other cancer types [38]). Overall, our study provides the first comprehensive analysis of the eligibility criteria in recent clinical trials in BC.

The study has two main limitations. Firstly, it included only trials registered with CT.gov and therefore some trials may have been missing from our analysis. However, so far CT.gov has been the largest registry of clinical trials and previous studies on the eligibility criteria used in oncology clinical trials were performed on data from CT.gov [16, 17, 19]. The other limitation is that we relied only on the data from CT.gov and did not have access to full trial protocols.

## Conclusions

In conclusion, the eligibility criteria in recent clinical trials in BC remain very restrictive; in fact, the frequency of some types of these criteria has significantly increased over the past 10 years. This seriously reduces the evidence base for the treatment of patients with certain characteristics commonly encountered in clinical practice. Therefore, the investigators should consider broadening the eligibility criteria. Our specific recommendations include the following: (1) Elimination of age limits; (2) More trials dedicated exclusively to older adults and patients with poor performance status; (3) Careful relaxation of some of the exclusion criteria concerning comorbidities including concurrent or prior malignancy. This will allow for the assessment of the safety and efficacy of anticancer drugs in populations of patients that have traditionally been underrepresented in oncology clinical trials.

## Supplementary Information

**Additional file 1.** List of included clinical trials in breast cancer.

**Additional file 2: Table S1.** Covariates affecting the odds of the presence of the upper age limits in clinical trials in breast cancer.

**Additional file 3: Table S2.** Covariates affecting the odds of the presence of strict exclusion criteria concerning comorbidities in clinical trials in breast cancer.

**Additional file 4: Table S3.** Covariates affecting the odds of the presence of the exclusion criteria involving the performance status of the patient in clinical trials in breast cancer.

## Data Availability

The datasets used and/or analyzed during the current study are available from the corresponding author on reasonable request.

## Abbreviations

ACS: American Cancer Society
aOR: Adjusted odds ratio
ASCO: American Society of Clinical Oncology
BC: Breast cancer
CGA: Comprehensive geriatric assessment
CI: Confidence interval
CT.gov: ClinicalTrials.gov
ECOG: Eastern Cooperative Oncology Group
FDA: Food and Drug Administration
IR: Interquartile range
NLM: National Library of Medicine
NIH: National Institutes of Health
OR: Odds ratio
WHO: World Health Organization
