# Supplementary material for: Eligibility criteria in clinical trials in breast cancer: a cohort study
Source: BMC Med. 2023 Jul 3;21:240. doi: 10.1186/s12916-023-02947-y (PMC10318672; doi:10.1186/s12916-023-02947-y)
Supplement: Supplementary file 1 — Additional file 1. List of included clinical trials in breast cancer. [file 12916_2023_2947_MOESM1_ESM.docx]

List of included clinical trials in breast cancer

|  | **NCT Number** | **Title** |
| --- | --- | --- |
| 1. | NCT05451784 | Treatment of Advanced or Metastatic Triple-negative Breast Cancer With Adoptive Therapy of PD1+ TILS |
| 2. | NCT05452213 | Comprehensive Analysis of Spatial, Temporal and Molecular Patters of Ribociclib Efficacy and Resistance in Advanced Breast Cancer Patients |
| 3. | NCT05447702 | Study of Camrelizumab Plus Apatinib and Chemotherapy as Neoadjuvant Therapy in Participants With Triple Negative Breast Cancer (TNBC) |
| 4. | NCT05444998 | Neoadjuvant Inituomab, Pyrrolidone and Nab-paclitaxel for HER2+Breast Cancer |
| 5. | NCT05445908 | SKB264 +/- KL-A167 in Recurrent or Metastatic Triple-negative Breast Cancer |
| 6. | NCT05438706 | A Clinical Study of the Efficacy and Safety of Chidamide in Combination With Camrelizumab and Carboplatin or Capecitabine in the Second and Third Line Treatment of Relapsed/Metastatic Triple-negative Breast Cancer |
| 7. | NCT05438810 | This is a Multicenter, Randomized, Double-blind, Placebo-controlled Phase III Clinical Study Evaluating the Efficacy and Safety of FCN-437c in Combination With Fluvestrant ± Goseraline Versus Placebo Combined With Fulvestrant ± Goserelin in Women With HR+ and HER2- Advanced Breast Cancer. |
| 8. | NCT05439499 | This is a Multicenter, Randomized, Double-blind, Placebo-controlled Phase III Clinical Study Evaluating the Efficacy and Safety of FCN-437c Versus Placebo in Combination With Letrozole or Anastrozole ± Goserelin in Women With HR+ and HER2- Advanced Breast Cancer. |
| 9. | NCT05433480 | A Study of BPI-16350 in Combination With Fulvestrant in Patients With HR+ and HER2- Locally Advanced, Recurrent or Metastatic Breast Cancer |
| 10. | NCT05430347 | Clinical Study of Pyrotinib in Neoadjuvant Therapy of HR-positive and HER2-positive Breast Cancer |
| 11. | NCT05430399 | Utidelone Versus Docetaxel in HER2-negative Locally Advanced or Metastatic Breast Cancer |
| 12. | NCT05431504 | The Efficacy and Safety of Dalpiciclib Plus Endocrine Therapy in HR-positive / HER2-negative Advanced Breast Cancer Patients With Visceral Crisis |
| 13. | NCT05429294 | Novel Double Target Therapy Combined With Chemotherapy in First-line Treatment of HER2+ Breast Cancer, MA-BC-II-038 |
| 14. | NCT05429684 | Precise Therapy for Refractory HER2 Positive Advanced Breast Cancer |
| 15. | NCT05426486 | A Study of ARX788 Combined With Pyrotinib Maleate Versus TCBHP (Trastuzumab Plus Pertuzumab With Docetaxel and Carboplatin) as Neoadjuvant Treatment in HER2-positive Breast Cancer Patients |
| 16. | NCT05422794 | Testing the Addition of Anti-Cancer Drug, ZEN003694 (ZEN-3694) and PD-1 Inhibitor (Pembrolizumab), to Standard Chemotherapy (Nab-Paclitaxel) Treatment in Patients With Advanced Triple-Negative Breast Cancer |
| 17. | NCT05424835 | A Trial of SHR-A1811versus Pyrotinib in Combination With Capecitabine in HER2-Positive, Unresectable and/or Metastatic Breast Cancer Subjects Previously Treated With Trastuzumab and Taxane |
| 18. | NCT05420454 | A Study for the Neoadjuvant Treatment of Breast Cancer |
| 19. | NCT05420467 | A Study for the Adjuvant Treatment of Breast Cancer |
| 20. | NCT05420779 | A Study to Evaluate the Efficacy and Safety of TSL-1502 Capsules in Breast Cancer Patients With Germline BRCA Mutations |
| 21. | NCT05415215 | A Study to Evaluate Patient Preference for Home Administration of Fixed-Dose Combination of Pertuzumab and Trastuzumab for Subcutaneous Administration in Participants With Early or Locally Advanced/Inflammatory HER2-Positive Breast Cancer |
| 22. | NCT05411380 | Tucidinosta Combined With Metronomic Capecitabine and Endocrine Therapy for Advanced HR-positive, HER2-negative Breast Cancer After CDK4/6 Inhibitor. |
| 23. | NCT05403333 | Weekly Utidelone in HER2-negative Inoperable Locally Advanced or Metastatic Breast Cancer |
| 24. | NCT05400993 | Clinical Study of Chidamide Combined With Chemotherapy in Neoadjuvant Treatment of HR+/ HER2-BC |
| 25. | NCT05402722 | Study of Eribulin in Combination With Anti-PD-1 Antibody in Patients With Metastatic Triple-Negative Breast Cancer |
| 26. | NCT05398861 | Utidelone Combined With Bevacizumab in the Treatment of ≥ 2 Lines of HER-2 Negative Advanced Breast Cancer |
| 27. | NCT05392608 | SEQUence of Endocrine Therapy in Advanced Luminal Breast Cancer (SEQUEL-Breast) |
| 28. | NCT05390476 | Tucidinostat and Metronomic Capecitabine for Metastatic Triple-negative Breast Cancer:a Multicenter,Open-label, Randomized Controlled, Phase II Clinical Trial |
| 29. | NCT05388149 | Kadcyla And Neratinib for Interception of HER2+ Breast Cancer With Molecular Residual Disease |
| 30. | NCT05388500 | Protocol for Herceptin as Adjuvant Therapy With Reduced Exposure to Chemotherapy (PHARE-C) |
| 31. | NCT05385705 | A Study of Allogenic Natural Killer Cells in Combination With Trastuzumab and Pertuzumab in Adult Patients With Refractory Metastatic Her2 Positive Breast Cancer. NK-ACT-BC_2020 |
| 32. | NCT05386108 | Study of Abemaciclib and Elacestrant in Patients With Brain Metastasis Due to HR+/Her2- Breast Cancer |
| 33. | NCT05386524 | Sintilimab and Bevacizumab Biosimilar Combined With PLD in mTNBC |
| 34. | NCT05383196 | Phase 1b/2 Study of Onvansertib + Paclitaxel in Triple-Negative Breast Cancer (TNBC) |
| 35. | NCT05384119 | Study of TTI-101 in Combination for Participants With Metastatic Hormone Receptor-Positive and Human Epithelial Receptor 2 (HER2)-Negative Breast Cancer |
| 36. | NCT05382286 | Study of Sacituzumab Govitecan-hziy and Pembrolizumab Versus Treatment of Physician's Choice and Pembrolizumab in Patients With Previously Untreated, Locally Advanced Inoperable or Metastatic Triple-Negative Breast Cancer |
| 37. | NCT05382299 | Study of Sacituzumab Govitecan-hziy Versus Treatment of Physician's Choice in Patients With Previously Untreated Metastatic Triple-Negative Breast Cancer |
| 38. | NCT05378464 | Adoptive T Cell Therapy Following HER2-Pulsed Dendritic Cell Vaccine & Pepinemab /Trastuzumab in Patients w/ Metastatic HER2+ Breast Cancer |
| 39. | NCT05376527 | Open Multi-cohort Study of the First Phase of Safety of a Drug Based on Double Recombinant Vaccinia Virus VV-GMCSF-Lact |
| 40. | NCT05374512 | A Study of Dato-DXd Versus Investigator's Choice Chemotherapy in Patients With Locally Recurrent Inoperable or Metastatic Triple-negative Breast Cancer, Who Are Not Candidates for PD-1/PD-L1 Inhibitor Therapy (TROPION-Breast02) |
| 41. | NCT05375461 | TQB3616 Capsules Plus Fulvestrant Compared to Placebo Plus Fulvestrant in Previously Treated Breast Cancer in Clinical Trail |
| 42. | NCT05365178 | To Evaluate the Efficacy and Safety of TQB3616 in Combination With Flulvesant Versus Placebo in Combination With Flulvesant in Previously Untreated Hormone-receptor (HR)-Positive, Human Epidermal Growth Factor Receptor 2 (HER2)-Negative Advanced Breast Cancer |
| 43. | NCT05362760 | Combination of Abemaciclib and Endocrine Therapy in Hormone Receptor Positive HER2 Negative Locally Advanced or Metastatic Breast Cancer With Focus on Digital Side Effect Management |
| 44. | NCT05357417 | Utidelone Plus Bevacizumab for Advanced Breast Cancer With Brain Metastases |
| 45. | NCT05353361 | Ib/II Phase Study of SHR-A1811 Injection in HER2 Positive Breast Cancer |
| 46. | NCT05346107 | PLD-cyclophosphamide-Nab-P Continuously Combined With Dual HER2 Blockage for HER2-positive Breast Cancer |
| 47. | NCT05346224 | A Study to Evaluate the Efficacy and Safety of HLX11 vs. EU-Perjeta® in the Neoadjuvant Therapy of HER2-Positive and HR-Negative Early-stage or Locally Advanced Breast Cancer |
| 48. | NCT05346328 | HER2-positive Breast Cancer Project Initiated by Investigators |
| 49. | NCT05346861 | Pyrotinib Rechallenge in Her2-positive Metastatic Breast Cancer Pretreated With Pyrotinib and Trastuzumab |
| 50. | NCT05347134 | SKB264 Injection vs Investigator Selected Regimens to Treat Locally Advanced, Recurrent or Metastatic Triple-negative Breast Cancer |
| 51. | NCT05336721 | A Phase II Study of Chiauranib in Combine With Capecitabine in TNBC |
| 52. | NCT05337657 | A Phase Ib Study of GB491 Plus Letrozole in Patients With Advanced Breast Cancer |
| 53. | NCT05334810 | DP303c in Patients With HER2-positive Unresectable Locally Advanced, Relapsed, or Metastaticbreast Cancer |
| 54. | NCT05335473 | Eribulin Plus Tucidinostat in the Treatment of HER2-negative Advanced Breast Cancer |
| 55. | NCT05332561 | Genomics Guided Targeted Post-neoadjuvant Therapy in Patients With Early Breast Cancer (COGNITION-GUIDE) |
| 56. | NCT05333328 | OFS in Premenopausal Node+ Breast Cancer With Low Genomic Risk |
| 57. | NCT05331326 | A Study of RC48-ADC for the Treatment of HER2-expression Metastatic Breast Cancer With Abnormal Activation of PAM Pathway |
| 58. | NCT05328440 | Treatment of Dalpiciclib Combined With Pyrotinib for Trastuzumab-sensitive HER2+ Advanced Breast Cancer(DAP-Her-02) |
| 59. | NCT05325632 | Study of HER2 Directed Dendritic Cell (DC1) Vaccine + Weekly Paclitaxel, Trastuzumab & Pertuzumab |
| 60. | NCT05323955 | Secondary BRain Metastases Prevention After Isolated Intracranial Progression on Trastuzumab/Pertuzumab or T-DM1 in Patients With aDvanced Human Epidermal Growth Factor Receptor 2+ brEast Cancer With the Addition of Tucatinib |
| 61. | NCT05318469 | Ivermectin and Pembrolizumab for the Treatment of Metastatic Triple Negative Breast Cancer |
| 62. | NCT05319873 | Ribociclib, Tucatinib, and Trastuzumab for the Treatment of HER2 Positive Breast Cancer |
| 63. | NCT05306340 | A Study Evaluating the Efficacy and Safety of Giredestrant Plus Everolimus Compared With Exemestane Plus Everolimus in Participants With Estrogen Receptor-Positive, HER2-Negative, Locally Advanced or Metastatic Breast Cancer (evERA Breast Cancer) |
| 64. | NCT05302336 | AC vs TC in Patients With HR-positive, HER2-negative Early Breast Cancer |
| 65. | NCT05304962 | First-in-Human Study of RGT-419B Alone and With Endocrine Therapy in Subjects With HR-Positive, HER2-Negative Advanced/Metastatic Breast Cancer |
| 66. | NCT05305365 | Study Assessing QBS72S For Treating Brain Metastases of Triple Negative Breast Cancer |
| 67. | NCT05306041 | Neoadjuvant Endocrine Therapy +/- the PI3K Inhibitor Inavolisib in HER2+, HR+, PIK3CA Mutant Early Breast Cancer |
| 68. | NCT05300958 | Deferoxamine Plus Chemotherapy for Metastatic Triple Negative Breast Cancer |
| 69. | NCT05297617 | Deescalation of Endocrine Therapy Duration in Women With HR+ HER2- Breast Cancer at Very Low Risk |
| 70. | NCT05296577 | Anlotinib Combination With Vinorelbine in the HER2- Advanced Breast Cancer |
| 71. | NCT05296746 | Neoadjuvant and Adjuvant Ribociclib and ET for Clinically High-risk ER+ and HER2- Breast Cancer |
| 72. | NCT05296798 | A Study to Evaluate the Efficacy and Safety of Giredestrant in Combination With Phesgo (Pertuzumab, Trastuzumab, and Hyaluronidase-zzxf) Versus Phesgo in Participants With Locally Advanced or Metastatic Breast Cancer (heredERA Breast Cancer) |
| 73. | NCT05293964 | Phase I Study to Evaluate SCR-6852 Alone or in Combination in ER+, HER2- Locally Advanced or Metastatic Breast Cancer |
| 74. | NCT05291910 | Inetetamab Combined With Anti-PD-1 Monoclonal Antibody and Albumin-Bound Paclitaxel for HER2+ Metastatic Breast Cancer |
| 75. | NCT05292742 | Compare Continuation of Original Targeted Therapy With Trastuzumab Combined With Pyrotinib and Capecitabine as Postoperative Adjuvant Therapy in Non-pCR Patients With HER2 Positive Early Breast Cancer |
| 76. | NCT05288127 | Efficacy of Talazoparib in Asian Metastatic Breast Cancer Patients With a Homologous Recombinant Deficiency (HRD) Signature |
| 77. | NCT05288777 | Adjuvant Chemoradiation and Biomarkers of Response in High-risk Breast Cancer |
| 78. | NCT05286437 | Phase II Lenvatinib and Pembrolizumab in Endocrine Resistant Breast Cancer With Letrozole |
| 79. | NCT05283837 | A Study to Evaluate Safety, Efficacy, Pharmacokinetics, and Immunogenicity of Test Pertuzumab (ZRC-3277, Cadila Healthcare Ltd.,) |
| 80. | NCT05275777 | A Phase Ib Safety lead-in, Followed by Phase II Trial of ADG106 in Combination With Neoadjuvant Chemotherapy in HER2 Negative Breast Cancer |
| 81. | NCT05266105 | A Phase 1 Study of Oral OP-1250 in Combination With Palbociclib in HR+/HER2- Breast Cancer Patients |
| 82. | NCT05266937 | Atezolizumab Plus CArboplatin Plus Nab-paclitaxel |
| 83. | NCT05263869 | A Study of MRG002 in the Treatment of Patients With HER2-positive Breast Cancer With Liver Metastases. |
| 84. | NCT05263882 | Gemcitabine Combined With Eribulin Regimen in the Treatment of Second-line Above Recurrent HER2-negative Breast Cancer |
| 85. | NCT05261269 | A Dose-escalation Study of the Safety and Pharmacology of DAN-222 in Subjects With Metastatic Breast Cancer |
| 86. | NCT05257395 | A Study of XZP-3287 in Combination With Letrozole/Anastrozole in Patients With Advanced Breast Cancer |
| 87. | NCT05255666 | Combination Liposomal Irinotecan and Pembrolizumab For Triple-Negative Breast Cancer (TNBC) With Brain Metastases (BM) |
| 88. | NCT05251766 | Nab-paclitaxel Compared With Docetaxel in the Neoadjuvant Chemotherapy Breast Cancer |
| 89. | NCT05253066 | Chidamide Combined With Exemestane (+/- Goserelin) Versus Neoadjuvant Chemotherapy in Patients of Stage II-III HR-positive/HER2-negative Breast Cancer |
| 90. | NCT05243641 | Neratinib and Capmatinib Combination (Phase Ib/II) in Metastatic Breast Cancer and Inflammatory Breast Cancer Patients With Abnormal HER2 and c-Met Pathway Activity as Measured by the CELsignia Signaling Analysis Test |
| 91. | NCT05244993 | AK105 Plus Anlotinib Hydrochloride Combined With Albumin Paclitaxel as a First-line Therapy in Patients With Advanced Triple-negative Breast Cancer |
| 92. | NCT05233696 | Radiotherapy in Combo With Chemo and Immunotherapy in Patients With PD-L1 Positive Metastatic TNBC |
| 93. | NCT05230810 | Clinical Trial of Alpelisb and Tucatinib in Patients With PIK3CA-Mutant HER2+ Metastatic Breast Cancer. |
| 94. | NCT05232006 | PARP-inhibitor on Advanced Metastatic Breast Cancer in Germline PALB2 Mutations Carriers |
| 95. | NCT05228951 | Pyrotinib Maleate, Trastuzumab, SHR6390 and Letrozole in Combination for Stage II-III TPBC |
| 96. | NCT05227664 | A Study of AK117/AK112 in Metastatic Triple-Negative Breast Cancer |
| 97. | NCT05215106 | Short-term Pre-OPerative Durvalumab (MEDI 4736) in Early Small Triple Negative Breast Cancer Patients (POP-Durva) |
| 98. | NCT05212454 | Efficacy of Supplement Adjuvant Capecitabine in HR+/HER2- Breast Cancer Patients With High Risks |
| 99. | NCT05207514 | Compare the Efficacy and the Safety of Doxorubicin and Cyclophosphamide Followed by Taxotere Versus Doxorubicin and Cyclophosphamide Nanoxel M as Neoadjuvant Chemotherapy in Breast Cancer |
| 100. | NCT05207709 | Ribociclib vs. Palbociclib in Patients With Advanced Breast Cancer Within the HER2-Enriched Intrinsic Subtype |
| 101. | NCT05209529 | Chemo-free BRCA-targeted Neoadjuvant Strategy |
| 102. | NCT05205200 | Immune Therapy in HR-positive/HER2-negative Metastatic Breast Cancer(ENIGMA) |
| 103. | NCT05206656 | Safety and Efficacy of Eribullin or Eribulin Combined With Anlotinib in Metastatic Breast Cancer |
| 104. | NCT05203445 | A Study of Olaparib and Pembrolizumab in People With Triple Negative Breast Cancer (TNBC) or Hormone Receptor-positive HER2-negative Breast Cancer |
| 105. | NCT05198843 | Testing an Omega-3 Fatty Acid-Based Anti-Cancer Therapy for Patients With Triple-Negative Inflammatory Breast Cancer That Has Spread to Other Parts of the Body |
| 106. | NCT05191914 | Clinical Study of Fulvestrant Combined With Chidamide in the Treatment of Hormone Receptor-positive Advanced Breast Cancer Resistant to CDK4/6 Inhibitors |
| 107. | NCT05192798 | Albumin-Bound Paclitaxel Combined With Antiangiogenic Agents in First-line Treatment of Relapsed or Metastatic TNBC |
| 108. | NCT05190094 | Prediction of Treatment Efficacy of the Combination of Palbociclib/(Letrozole or Anastrozole) in First Line Metastatic Women With Luminal, HER2 Negative Advanced Breast Cancer, Using Infrared Laser Spectroscopy Analysis on Liquid Biopsies. |
| 109. | NCT05187832 | A Study of AND019 in Women With ER Positive HER2 Negative Advanced or Metastatic Breast Cancer |
| 110. | NCT05189067 | Efficacy and Safety of Adjuvant Docetaxel and Trastuzumab in Stage I HER2-positive Breast Cancer |
| 111. | NCT05189717 | A Trial of HRS-8080 Tablets in Metastatic or Local Advanced Breast Cancer of Adults |
| 112. | NCT05183828 | Effect of HSD3B1 (1245C) Gene Mutation on Treatment of Stage I-II Breast Cancer |
| 113. | NCT05186545 | An Exploratory Study of Surufatinib Combined With Chidamide and Fulvestrant in HR Positive Unresectable Metastatic Breast Cancer |
| 114. | NCT05183126 | Pharmacokinetic Study of Skeletal Muscle Area-based Paclitaxel Infusion in Patients With Breast Cancer |
| 115. | NCT05180006 | Impact of Neoadjuvant Immunotherapy in Early Stage Breast Cancer Before Standard Therapy |
| 116. | NCT05181033 | Lenvatinib+Letrozole Versus Fulvestrant in Metastatic ER+/HER2- Breast Cancer, Post Progression on Al + CDK4/6 Inhibitor |
| 117. | NCT05181462 | Nadunolimab in Combination With Gemcitabine Plus Carboplatin in Patients With Advanced Triple Negative Breast Cancer. |
| 118. | NCT05177796 | Panitumumab and Pembrolizumab in Combination With Neoadjuvant Chemotherapy for the Treatment of Stage III-IV Triple Negative Breast Cancer |
| 119. | NCT05176080 | Famitinib Plus SHR6390 and Endocrine Therapy in the Treatment of HR-positive, HER2-negative Advanced Breast Cancer |
| 120. | NCT05174832 | Induction of Cisplatin/Nab-paclitaxel/Pembrolizumab Followed by Olaparib/Pembrolizumab Maintenance in mTNBC Patients |
| 121. | NCT05172518 | Utidelone Plus Capecitabine Versus Taxane Plus Capecitabine in HER2-negative Locally Advanced or Metastatic Breast Cancer |
| 122. | NCT05169567 | Abemaciclib (LY2835219) Plus Fulvestrant Compared to Placebo Plus Fulvestrant in Previously Treated Breast Cancer |
| 123. | NCT05165225 | Phase II Neoadjuvant Pyrotinib Combined With Neoadjuvant Chemotherapy in HER2-low-expressing and HR Positive Early or Locally Advanced Breast Cancer: a Single-arm, Non-randomized, Single-center, Open Label Trial |
| 124. | NCT05163106 | Neoadjuvant Treatment of Locally-advanced Breast Cancer Patients With Ribociclib and Letrozole |
| 125. | NCT05163223 | Therapeutic Cancer Vaccine (AST-301, pNGVL3-hICD) in Patients With Breast Cancer |
| 126. | NCT05161195 | Roll-over Study to Allow Continued Access to Ribociclib |
| 127. | NCT05159193 | Neoadjuvant Treatment Pegylated Liposomal Doxorubicin Plus Cyclophosphamide Sequential Docetaxel Plus Trastuzumab and Pertuzumab Versus Docetaxel Plus Carboplatin Combined With Trastuzumab and Pertuzumab in HER-2 Positive Breast Cancer |
| 128. | NCT05159778 | Phase 2 Study of Imprime PGG and Pembrolizumab in Patients With HR+/HER2- Metastatic Breast Cancer (mBCA) |
| 129. | NCT05160545 | A Study of GNC-035, a Tetra-specific Antibody, in Participants With Locally Advanced or Metastatic Breast Cancer |
| 130. | NCT05154396 | Neratinib Dose Escalation Regimen for HER2 Positive Early Breast Cancer |
| 131. | NCT05150652 | Neoadjuvant Endocrine Therapy in ER-positive, HER2-negative Early Stage Breast Cancer |
| 132. | NCT05143229 | Alpelisib And Sacituzumab Govitecan For Treatment Of Breast Cancer |
| 133. | NCT05134519 | RC48 for Neoadjuvant Chemotherapy of HER2 Positive Breast Cancer |
| 134. | NCT05132582 | A Study of Tucatinib or Placebo With Trastuzumab and Pertuzumab for Metastatic HER2+ Breast Cancer |
| 135. | NCT05134194 | A Study of Camrelizumab in Combination With Chemotherapy Regimen Comparative Chemotherapy Regimen for Metastatic Triple-negative Breast Cancer |
| 136. | NCT05131841 | Cipterbin Combined With Vinorelbine in the Treatment of HER2-positive MBC |
| 137. | NCT05128734 | Temozolomide Monotherapy or in Combination With Olaparib in Patients With Triple Negative Breast Cancer (TNBC) |
| 138. | NCT05128773 | Study of Amcenestrant (SAR439859) Versus Tamoxifen for Patients With Hormone Receptor-positive (HR+) Early Breast Cancer, Who Have Discontinued Adjuvant Aromatase Inhibitor Therapy Due to Treatment-related Toxicity |
| 139. | NCT05122494 | A Phase Ⅲ Study of the Efficacy and Safety of Hemay022+Aromatase Inhibitor(AI) in Participants With ER+/HER2+ Advanced or Metastatic Breast Cancer |
| 140. | NCT05114720 | Moxifloxacin in Adjuvant Treatment of Patients With Operable Breast Cancer |
| 141. | NCT05112536 | Trilaciclib, a CDK4/6 Inhibitor, in Patients With Early-Stage Triple Negative Breast Cancer |
| 142. | NCT05113251 | Trastuzumab Deruxtecan (T-DXd) Alone or in Sequence With THP, Versus Standard Treatment (ddAC-THP), in HER2-positive Early Breast Cancer |
| 143. | NCT05113966 | Trilaciclib in Patients Receiving Sacituzumab Govitecan-hziy for Triple Negative Breast Cancer |
| 144. | NCT05110170 | Efficacy and Safety of LY01005 in Patients With Breast Cancer Compared to ZOLADEX® |
| 145. | NCT05104866 | A Phase-3, Open-Label, Randomized Study of Dato-DXd Versus Investigator's Choice of Chemotherapy (ICC) in Participants With Inoperable or Metastatic HR-Positive, HER2-Negative Breast Cancer Who Have Been Treated With One or Two Prior Lines of Systemic Chemotherapy (TROPION-Breast01) |
| 146. | NCT05103826 | A Study of SHR6390 Combined With Famitinib in the Treatment of ER + / HER2- Advanced Breast Cancer |
| 147. | NCT05103917 | A Study to Assess the Safety, Tolerability and Antitumor Activity of X4P-001 in Combination With TNBC |
| 148. | NCT05101564 | Umbrella Trial Testing Integrative Subtype-Targeted Therapeutics in HR+ /HER2-Negative Breast Cancer |
| 149. | NCT05097248 | Camrelizumab in Combination With PLD and Losartan in Patients With TNBC Who Have Received ≦ 1 Line of Chemotherapy |
| 150. | NCT05095207 | Abemaciclib in Combination With Bicalutamide for Androgen Receptor-positive, HER2-negative Metastatic Breast Cancer |
| 151. | NCT05093387 | SGT-53, Carboplatin, and Pembrolizumab for the Treatment of Metastatic Triple Negative Inflammatory Breast Cancer |
| 152. | NCT05088057 | Neoadjuvant Camrelizumab Plus Chemotherapy in Triple Negative Breast Cancer |
| 153. | NCT05085002 | A Study of Lerociclib in Participants With Advanced Breast Cancer |
| 154. | NCT05085626 | Fluzoparib in Combination With Chidamide or Camrelizumab for HRD Positive HER2 Negative Advanced Breast Cancer |
| 155. | NCT05080842 | A Study of AC682 for the Treatment of Locally Advanced or Metastatic ER+ Breast Cancer |
| 156. | NCT05081492 | CF33-hNIS-antiPDL1 for the Treatment of Metastatic Triple Negative Breast Cancer |
| 157. | NCT05079360 | Efficacy Evaluation of Sabizabulin Monotherapy Versus Active Control for Treatment of ER+HER2- Metastatic Breast Cancer |
| 158. | NCT05077449 | A Study of XZP-3287 in Combination With Fulvestrant in Patients With Advanced Breast Cancer |
| 159. | NCT05076682 | Reverse Triple Negative Immune Resistant Breast Cancer |
| 160. | NCT05076695 | Neoadjuvant With Trastuzumab, Pyrotinib Plus Palbociclib and Fulvestrant in HER2-positive, ER-positive Breast Cancer |
| 161. | NCT05074290 | Pharmacokinetics and Safety of Epidiferphane™ and Taxanes in Breast Cancer Patients |
| 162. | NCT05075512 | The Efficacy and Safety of Anlotinib Combined With Fulvestrant in Patients With Advanced Breast Cancer |
| 163. | NCT05069038 | Clinical Trial Assessing the Safety of Neoadjuvant Palbociclib in Combination With Endocrine Therapy |
| 164. | NCT05067530 | Cyclin dEpendent Kinase in tRiple nEGatIVe brEast canceR - a "Window of Opportunity" Study |
| 165. | NCT05068141 | A Study to Evaluate the Efficacy and Safety of SG001 in Combination With Nab-Paclitaxel in Patients With Advanced Triple-Negative Breast Cancer (TNBC) |
| 166. | NCT05065411 | Efficacy & Safety Evaluation of Enobosarm in Combo With Abemaciclib in Treatment of ER+HER2- Metastatic Breast Cancer |
| 167. | NCT05063786 | Trial of Trastuzumab + ALpelisib +/- Fulvestrant vs Trastuzumab + Chemotherapy in Patients With PIK3CA Mutated Previously Treated HER2+ Advanced BrEasT Cancer (ALPHABET) |
| 168. | NCT05064085 | Capecitabine In Combination With Cemiplimab In Patient With Metastatic Breast Cancer |
| 169. | NCT05054751 | GB491 Combined With Fulvestrant for HR+ HER2- Locally Advanced or Metastatic Breast Cancer |
| 170. | NCT05044988 | A Study of HS-10342 in Chinese Patients With HR+/HER2- Advanced and/or Metastatic Breast Cancer |
| 171. | NCT05041842 | Treatment With Tucatinib in Patients With an Isolated Brain Progression of a Metastatic Breast Cancer |
| 172. | NCT05042791 | A Study of Pyrotinib Plus Capecitabine Combined With SRT in HER2+ MBC With Brain Metastases |
| 173. | NCT05041101 | Grapiprant and Eribulin for the Treatment of Metastatic Inflammatory Breast Cancer |
| 174. | NCT05038735 | Study to Assess the Efficacy and Safety of Alpelisib Plus Fulvestrant in Participants With HR-postitive (HR+), HER2-negative, Advanced Breast Cancer After Treatment With a CDK4/6 Inhibitor and an Aromatase Inhibitor. |
| 175. | NCT05033756 | Comprehensive Analysis of Predictors of the Treatment With Pembrolizumab and Olaparib in Patients With Unresectable or Metastatic HER2 Negative Breast Cancer and a Deleterious Germline Mutation or a Homologous Recombination Deficiency (COMPRENDO |
| 176. | NCT05033769 | Assessing ImmunoResponse Post Eribulin: Eribulin and Immunogenicity in Advanced Breast Cancer |
| 177. | NCT05035836 | A Phase 2 Single-Arm Open-Label Pilot Trial Evaluating Zanidatamab (ZW25) in Patients With Early Stage HER2/Neu Positive (HER2+) Breast Cancer (BC) |
| 178. | NCT05036005 | Neoadjuvant Ontruzant (SB3) in Patients With HER2-positive Early Breast Cancer: An Open-Label (NeoON) |
| 179. | NCT05029999 | CD40 Agonist, Flt3 Ligand, and Chemotherapy in Triple Negative Breast Cancer |
| 180. | NCT05025735 | Alpelisib, Fulvestrant and Dapagliflozin for the Treatment of HR+, HER2 -, PIK3CA Mutant Metastatic Breast Cancer |
| 181. | NCT05021900 | Efficacy and Safety of Tenalisib (RP6530), a PI3K δ/γ and SIK3 Inhibitor, in Patients With Locally Advanced or Metastatic Breast Cancer |
| 182. | NCT05019690 | Apatinib Combined With Albumin-Bound Paclitaxel for Treatment of Advanced Triple Negative Breast Cancer |
| 183. | NCT05020860 | Correlation of Clinical Response to Pathologic Response in Patients With Early Breast Cancer |
| 184. | NCT05020964 | Screening Plasma HER2 Amplication by Digital PCR in Advanced Breast Cancer Patients Treated With Trastuzumab and Pyrotinib |
| 185. | NCT05018676 | ARX788 in Breast Cancer With Low Expression of HER2 |
| 186. | NCT05018702 | ARX788 in HER2-positive Breast Cancer Patients With Brain Metastases |
| 187. | NCT05016349 | Investigating the Potential Role of a Novel Quadrate Combination Therapy Mifepristone(Antiprogestrone), Tamoxifen, Retinoic Acid and Cannabidiol ( Selective Cyp 26 Inhibitor) for Treating Early Breast Cancer. |
| 188. | NCT05007444 | Safety and Efficacy Evaluation of the P2Et Extract in Patients With Breast Cancer |
| 189. | NCT05004142 | Study of Efficacy, Safety, and Pharmacokinetics of FCN-437c in Combination With Fulvestrant or Letrozole+Goserelin |
| 190. | NCT04999540 | Tucidinostat and Fulvestrant in Hormone-receptor Positive Advanced Breast Cancer |
| 191. | NCT04997798 | Neoadjuvant Study of Dalpiciclib in Combination With Letrozole and Trastuzumab Plus Pyrotinib in Early ER+ HER2+ Breast Cancer |
| 192. | NCT04997941 | Higher Dose Preoperative taMOxifen in Premenopausal bREast Cancer Patients |
| 193. | NCT04993014 | Circulating Tumor Cells and Treatment De-escalation After Neoadjuvant Therapy for HER2 Positive Breast Cancer |
| 194. | NCT04993430 | A Phase 1 Study of HRS8807 Monotherapy and in Combination With SHR6390 in Subjects With ER-Positive, HER2-Negative Metastatic or Locally Advanced Breast Cancer |
| 195. | NCT04990921 | Study of Palliative Radiation Combined With Pembrolizumab in Unresectable Metastatic Stage IV Breast Cancer |
| 196. | NCT04986852 | Olinvacimab With Pembrolizumab in Patients With mTNBC |
| 197. | NCT04985266 | A Trial of Early Detection of Molecular Relapse With Circulating Tumour DNA Tracking and Treatment With Palbociclib Plus Fulvestrant Versus Standard Endocrine Therapy in Patients With ER Positive HER2 Negative Breast Cancer |
| 198. | NCT04983121 | Efficacy and Safety of Pyrotinib Maleate Combined With ARX788 Neoadjuvant Treatment in Breast Cancer Patients |
| 199. | NCT04975308 | A Study of Imlunestrant, Investigator's Choice of Endocrine Therapy, and Imlunestrant Plus Abemaciclib in Participants With ER+, HER2- Advanced Breast Cancer |
| 200. | NCT04975451 | ADCb (Anlotinib/Docetaxel/Carboplatin) as Neoadjuvant Therapy for Triple-Negative Breast Cancer |
| 201. | NCT04973319 | Trastuzumab Combined With Pertuzumab for Adjuvant Treatment of Breast Cancer After Neoadjuvant Therapy |
| 202. | NCT04964934 | Phase III Study to Assess AZD9833+ CDK4/6 Inhibitor in HR+/HER2-MBC With Detectable ESR1m Before Progression (SERENA-6) |
| 203. | NCT04965064 | Trial of Neratinib Plus Capecitabine in Subjects With HER2-Negative Metastatic Breast Cancer With Brain Metastases and Abnormally Active HER2 Signaling |
| 204. | NCT04965688 | Systems Biology Guided Therapy for Breast Cancer Positive for Oestrogen Receptor After Aromatase Inhibitor and CDK Inhibition |
| 205. | NCT04965766 | Patritumab Deruxtecan (U3-1402) in Unresectable Locally Advanced or Metastatic Breast Cancer |
| 206. | NCT04963595 | Pyrotinib and Vinorelbine Tartrate Capsules With or Without Inetetamab for First Line Treatment in Patients With Trastuzumab-resistant HER2-positive Advanced Breast Cancer |
| 207. | NCT04961632 | Dose-determining and Dose-confirmatory Study to Investigate the Optimal Dose of Tamoxifen in Breast Cancer Patients According to Genotype Status of TCF20 rs932376 |
| 208. | NCT04961996 | A Study Evaluating the Efficacy and Safety of Adjuvant Giredestrant Compared With Physician's Choice of Adjuvant Endocrine Monotherapy in Participants With Estrogen Receptor-Positive, HER2-Negative Early Breast Cancer (lidERA Breast Cancer) |
| 209. | NCT04958785 | Study to Evaluate the Safety and Efficacy of Magrolimab Combination Therapy in Adults With Unresectable, Locally Advanced or Metastatic Triple-Negative Breast Cancer |
| 210. | NCT04946864 | A Study to Investigate the Safety, Tolerability, of APG-2575 as a Single Agent or in Combination for Breast Cancer |
| 211. | NCT04947189 | Seviteronel in Combination With Chemotherapy in Androgen-receptor Positive Metastatic Triple-negative Breast Cancer |
| 212. | NCT04946227 | Pembrolizumab and Paclitaxel in Hormone Receptor-positive, hyperMUTATted Metastatic Breast Cancer Identified by Whole exOme sequeNcing ('MUTATION2') |
| 213. | NCT04941885 | Inetetamab Plus Cyclophosphamide Metronomic Chemotherapy Plus Aromatase Inhibitor in Metastatic HER2+/HR+ Breast Cancer |
| 214. | NCT04942054 | A Study in Patients With Advanced Breast Cancer |
| 215. | NCT04936295 | Fulvestrant Plus Anlotinib in HR(+)/HER2(-) Metastatic Breast Cancer With FGFR Mutation |
| 216. | NCT04929548 | Exploratory Study of Neoadjuvant Treatment of HER2-positive Breast Cancer With Py in Combination With HP |
| 217. | NCT04927481 | A Study of Mitoxantrone Hydrochloride Liposome Injection in Patients With Advanced HER2 Negative Breast Cancer |
| 218. | NCT04927884 | A Study of Sacituzumab With Chemoimmunotherapy to Treat Advanced Triple-Negative Breast Cancer After Prior Therapies |
| 219. | NCT04928261 | Evaluating 6-months of HER2-targeted Therapy in Patients With HER2 Positive Early-stage Breast Cancer That Achieve a Pathological Complete Response to Neoadjuvant Systemic Therapy |
| 220. | NCT04924699 | A Study of MRG002 in the Treatment of Patients With HER2-positive Unresectable Locally Advanced or Metastatic Breast Cancer. |
| 221. | NCT04923542 | Stereotactic Radiation & Abemaciclib in the Management of HR+/HER2- Breast Cancer Brain Metastases |
| 222. | NCT04920708 | Fulvestrant, Ipatasertib and CDK4/6 Inhibition in Metastatic ER+/HER2- Breast Cancer Patients Without ctDNA Suppression |
| 223. | NCT04922008 | Stage I HER2 Positive Invasive Breast Cancer De-escalation Study(IRIS-C/D) |
| 224. | NCT04917900 | Single-arm, Multi-center Clinical Study of Pyrotinib Maleate Tablets Combined With Albumin-bound Paclitaxel and Trastuzumab in Neoadjuvant Treatment of Her2-positive Early or Locally Advanced Breast Cancer |
| 225. | NCT04915755 | Efficacy and Safety Comparison of Niraparib to Placebo in Participants With Human Epidermal Growth Factor 2 Negative (HER2-) Breast Cancer Susceptibility Gene Mutation (BRCAmut) or Triple-Negative Breast Cancer (TNBC) With Molecular Disease |
| 226. | NCT04913571 | Tislelizumab in Combination With Eribulin for Patients With Metastatic Previously heaviLy-treAted TriplE-negative Breast Cancer：A Prospective Multiple-center Phase II Study |
| 227. | NCT04914390 | A Phase Ⅱ Study of Anlotinib Combined With Tislelizumab and AT in the Neoadjuvant Treatment of Triple-negative Breast Cancer |
| 228. | NCT04906395 | Ovarian Suppression Evaluating Subcutaneous Leuprolide Acetate in Breast Cancer |
| 229. | NCT04907344 | Study of Camrelizumab Plus Chemotherapy as Neoadjuvant Therapy in Participants With Triple Negative Breast Cancer (TNBC) |
| 230. | NCT04903652 | Pyrotinib Maleate Combined With Vinorelbine in the Treatment of HER2-positive Advanced Breast Cancer |
| 231. | NCT04899908 | Stereotactic Brain-directed Radiation With or Without Aguix Gadolinium-Based Nanoparticles in Brain Metastases |
| 232. | NCT04900311 | Pyrotinib Versus Pertuzumab in Combination With Neoadjuvant Trastuzumab and Nab-Paclitaxel in HER2+ Early or Locally Advanced Breast Cancer |
| 233. | NCT04901299 | Fulvestrant + Neratinib In Breast Cancer |
| 234. | NCT04896320 | Tucatinib With Chemotherapy and Trastuzumab in Advanced Her-2-neu Overexpressing, Previously Treated Breast Cancer. |
| 235. | NCT04895358 | Study of Pembrolizumab (MK-3475) Plus Chemotherapy Versus Placebo Plus Chemotherapy for HR+/HER2- Locally Recurrent Inoperable or Metastatic Breast Cancer (MK-3475-B49/KEYNOTE-B49) |
| 236. | NCT04895761 | Neoadjuvant DPX-Survivac Aromatase Inhibition, Radiotherapy or Cyclophosphamide in HR+HER2- Breast Cancer |
| 237. | NCT04892693 | Talazoparib in Advanced Breast Cancer Patients With Homologous Recombinant Deficiency |
| 238. | NCT04893109 | ATEMPT 2.0: Adjuvant T-DM1 vs TH |
| 239. | NCT04891068 | BRE-04: Window of Opportunity Trial of Preoperative Low Dose Azacitidine in High-Risk Early Stage Breast Cancer |
| 240. | NCT04886531 | Trial of Pre-operative Neratinib and Endocrine Therapy With Trastuzumab in Triple Positive Breast Cancers |
| 241. | NCT04881929 | Study of KN026 in Combination With Docetaxel as Neoadjuvant Therapy in HER2-positive Breast Cancer |
| 242. | NCT04877821 | The Efficacy and Safety of Sintilimab Plus Anlotinib Combined With Chemotherapy as Neoadjuvant Therapy in TNBC |
| 243. | NCT04874935 | The Possible Efficacy and Safety of Lansoprazole Co-administration With Neoadjuvant Chemotherapy in Women With Breast Cancer |
| 244. | NCT04872985 | Pyrotinib in Combination With Neoadjuvant Chemotherapy in HR+/HER2-, HER4 High Expression Breast Cancer Patients: A Phase II Trial |
| 245. | NCT04873362 | A Study Evaluating the Efficacy and Safety of Adjuvant Atezolizumab or Placebo and Trastuzumab Emtansine for Participants With HER2-Positive Breast Cancer at High Risk of Recurrence Following Preoperative Therapy |
| 246. | NCT04872608 | A Study of Letrozole, Palbociclib, and Onapristone ER in People With Metastatic Breast Cancer |
| 247. | NCT04869943 | Efficacy Evaluation of Enobosarm Monotherapy in Treatment of AR+/ER+/HER2- Metastatic Breast Cancer |
| 248. | NCT04862663 | Capivasertib + Palbociclib + Fulvestrant for HR+/HER2- Advanced Breast Cancer (CAPItello-292). |
| 249. | NCT04862143 | Pilot Decentralized Clinical Trial in Men and Pre and Post-menopausal Women With Breast Cancer and a Specific Mutation (PIK3CA) Treated With Alpelisib in Combination With Fulvestrant |
| 250. | NCT04858516 | Neoadjuvant Treatment With Palbociclib and Exemestane Plus Trastuzumab and Pyrotinib in Estrogen Receptor (ER)-Positive, HER2-positive Breast Cancer |
| 251. | NCT04858529 | Neoadjuvant TCHP Versus THP in Patients With HER2-positive Breast Cancer (neoCARHP Study) |
| 252. | NCT04858997 | Tumor Response Time of Palbociclib in Combination With AI in Real-world Chinese Patients |
| 253. | NCT04856371 | Study of CYH33 in Combination With Endocrine Therapy With or Without Palbociclib in Patients With HR+, HER2- Advanced Breast Cancer |
| 254. | NCT04852419 | A PHASE 1B STUDY OF ZN-C5 IN CHINESE SUBJECTS |
| 255. | NCT04852887 | De-Escalation of Breast Radiation Trial for Hormone Sensitive, HER-2 Negative, Oncotype Recurrence Score Less Than or Equal to 18 Breast Cancer (DEBRA) |
| 256. | NCT04851613 | Study Evaluating Efficacy & Safety of Afuresertib Plus Fulvestrant in Patients w/ Locally Advanced or Metastatic HR+/HER2- Breast Cancer |
| 257. | NCT04848454 | Efficacy and Safety of Combinition of Camrelizumab in Second-line Neoadjuvant Chemotherapy and Adjuvant Therapy |
| 258. | NCT04849364 | Circulating Tumor DNA Enriched, Genomically Directed Post-neoadjuvant Trial for Patients With Residual Triple Negative Breast Cancer |
| 259. | NCT04842617 | Phase III Study ：SHR6390/Placebo Combined With Endocrine Therapy for the Adjuvant Treatment of Hormone Receptor Positive,Human Epidermal Receptor 2 Negative |
| 260. | NCT04841148 | Avelumab or Hydroxychloroquine With or Without Palbociclib to Eliminate Dormant Breast Cancer |
| 261. | NCT04836156 | Neoadjuvant Therapy Study Guided by Drug Screening in Vitro for Human Epidermal Growth Factor Receptor 2 (HER2) Negative Early Breast Cancer Patients |
| 262. | NCT04836520 | SHR6390 Combined With Anastrozole for Preoperative Treatment of Breast Cancer. |
| 263. | NCT04837209 | Radiation, Immunotherapy and PARP Inhibitor in Triple Negative Breast Cancer |
| 264. | NCT04829604 | ARX788 in HER2-positive, Metastatic Breast Cancer Subjects (ACE-Breast-03) |
| 265. | NCT04826016 | POL6326 (Balixafortide) Plus Nab-paclitaxel or Eribulin in Patients With HER2-negative Advanced Breast Cancer |
| 266. | NCT04817540 | Phase II Trial of Anti-HER2 Treatment in HER2-enriched Early Breast Cancer Identified by PAM50 (HER2E-PAM, PAMILIA Study) |
| 267. | NCT04818632 | AZD9833 China PK Study |
| 268. | NCT04819243 | PALbociclib Endocrine Therapy Followed by Talazo vs. Talazoz-Atezo Study |
| 269. | NCT04809779 | PD-1 Inhibitor Concurrent With Chemotherapy as Neoadjuvant Therapy for TNBC |
| 270. | NCT04802759 | A Study Evaluating the Efficacy and Safety of Multiple Treatment Combinations in Participants With Breast Cancer |
| 271. | NCT04803539 | A Prospective, Phase II Trial Using ctDNA to Initiate Post-operation Boost Therapy After Adjuvant Chemotherapy in TNBC |
| 272. | NCT04799249 | Trilaciclib, a CDK 4/6 Inhibitor, in Patients Receiving Gemcitabine and Carboplatin for Metastatic Triple-Negative Breast Cancer (TNBC) |
| 273. | NCT04796623 | A Study of TQB3616 Capsules Combined With Fulvestrant Injection in Subjects With Advanced Breast Cancer |
| 274. | NCT04796324 | Anti-tumor Effect of Ixabepilone in Metastatic Breast Cancer (mBC) Selected by the Ixabepilone DRP. |
| 275. | NCT04790305 | Effect of Huaier Granule on Adjuvant Treatment for High-risk Early-stage Triple-negative Breast Cancer |
| 276. | NCT04791384 | Phase Ib/II Trial of Abemaciclib and Elacestrant in Patients With Brain Metastasis Due to HR+/Her2- Breast Cancer |
| 277. | NCT04789096 | Tucatinib Together With Pembrolizumab and Trastuzumab |
| 278. | NCT04784715 | Trastuzumab Deruxtecan (T-DXd) With or Without Pertuzumab Versus Taxane, Trastuzumab and Pertuzumab in HER2-positive Metastatic Breast Cancer (DESTINY-Breast09) |
| 279. | NCT04780347 | Albumin-bound Paclitaxel Combination With Capecitabine Versus Capecitabine Monotherapy in Paclitaxel/Docetaxel-resistant Advanced Breast Cancer |
| 280. | NCT04770272 | Study to Compare a Mono Atezolizumab Window Followed by a Atezolizumab - CTX Therapy With Atezolizumab - CTX Therapy |
| 281. | NCT04771871 | MicroRNA Profiles in Triple Negative Breast Cancer |
| 282. | NCT04768426 | Serial Circulating Tumor DNA (ctDNA) Monitoring During Adjuvant Capecitabine in Early Triple-negative Breast Cancer |
| 283. | NCT04767828 | A Single Arm Study of Brain Metastasis in Patients With HER2-positive Breast Cancer |
| 284. | NCT04762979 | Alpelisib (BYL719) in Combination With Continued Endocrine Therapy Following Progression on Endocrine Therapy in Hormone Receptor Positive, HER2 Negative, PIK3CA Mutant Metastatic Breast Cancer |
| 285. | NCT04759248 | Study With Atezolizumab in Combination With Trastuzumab and Vinorelbine in HER2-positive Advanced/Metastatic Breast Cancer |
| 286. | NCT04760431 | TKIs vs. Pertuzumab in HER2+ Breast Cancer Patients With Active Brain Metastases (HER2BRAIN) |
| 287. | NCT04755868 | Talazoparib Maintenance Therapy in Triple-negative Breast Cancer |
| 288. | NCT04756505 | Immunotherapy (NHS-IL12 & Bintrafusp Alfa) and Radiation Therapy for the Treatment of Hormone Receptor Positive, HER2 Negative Metastatic Breast Cancer, the REINA Trial |
| 289. | NCT04756765 | Talazoparib Monotherapy in PALB2 Mutation Associated Advanced Breast Cancer |
| 290. | NCT04753177 | The Neoadjuvant Combined Hormone Therapy in Premenopausal Women With Locally Advanced ER+/HER2- Breast Cancer |
| 291. | NCT04752059 | Phase II Study of T-DX in HER2-positive Breast Cancer Brain Metastases |
| 292. | NCT04752332 | A Study of Abemaciclib (LY2835219) Plus Hormone Therapy in Participants With Early Breast Cancer |
| 293. | NCT04750122 | Neoadjuvant Therapy Study Guided by Drug Screening in Vitro for HER2 Positive Early Breast Cancer Patients |
| 294. | NCT04745975 | Guided Treatment Based on Mini-PDX in Metastatic Triple Negative Breast Cancer |
| 295. | NCT04739670 | Evaluating the Efficacy and Safety of Bevacizumab, Carboplatin, Gemcitabine and Atezolizumab in Breast Cancer |
| 296. | NCT04739761 | A Study of T-DXd in Participants With or Without Brain Metastasis Who Have Previously Treated Advanced or Metastatic HER2 Positive Breast Cancer |
| 297. | NCT04740918 | A Study of Trastuzumab Emtansine in Combination With Atezolizumab or Placebo as a Treatment for Participants With Human Epidermal Growth Factor 2 (HER2)-Positive and Programmed Death-ligand 1 (PD-L1)-Positive Locally Advanced (LABC) or Metastatic Breast Cancer (MBC) |
| 298. | NCT04742153 | A Study of MRG002 in the Treatment of Patients With HER2-low Locally Advanced or Metastatic Breast Cancer (BC) |
| 299. | NCT04738292 | Onapristone and Fulvestrant for ER+ HER2- Metastatic Breast Cancer After Endocrine Therapy and CDK4/6 Inhibitors (The SMILE Study) |
| 300. | NCT04736589 | Inetetamab Plus Rapamycin and Chemotherapy for HER2+ Metastatic Breast Cancer With Abnormal Activation of PAM Pathway |
| 301. | NCT04733417 | A Study of SHR6390 Combined With Famitinib in the Treatment of HR + / HER2- Advanced Breast Cancer. |
| 302. | NCT04734262 | A Phase II Study to Explore the Safety, Tolerability, and Preliminary Antitumor Activity of Sitravatinib and Tislelizumab in Patients With Locally Recurrent or Metastatic Triple Negative Breast Cancer |
| 303. | NCT04732598 | A Phase III Study of Bevacizumab and Paclitaxel in Combination With Atezolizumab as a Treatment for Locally Advanced Unresectable or Metastatic Hormone Receptor-positive HER2 Negative Breast Cancer |
| 304. | NCT04733118 | Chemotherapy-Free pCR-Guided Strategy With Trastuzumab-pertuzumab and T-DM1 in HER2-positive Early Breast Cancer |
| 305. | NCT04728035 | Study of Irinotecan Liposome Injection in Patients With Advanced Breast Cance |
| 306. | NCT04721977 | A Study of Tucatinib (MK-7119) in Combination With Trastuzumab and Capecitabine in Participants With Previously Treated Locally Advanced Unresectable or Metastatic Human Epidermal Growth Factor Receptor 2 Positive (HER2+) Breast Carcinoma (MK-7119-001) |
| 307. | NCT04722718 | Efficacy and Safety of Sintilimab and Apatinib Combined Chemotherapy in Breast Cancer |
| 308. | NCT04722978 | Standard Chemotherapy Plus Moxifloxacin as First-line Treatment for Metastatic Triple-negative Breast Cancer |
| 309. | NCT04717531 | Pyrotinib as Neoadjuvant Agent for Non-objective Response HER2-positive Early Breast Cancer |
| 310. | NCT04720664 | Oral SM-88 in Patients With Metastatic HR+/HER2- Breast Cancer |
| 311. | NCT04714619 | CB-103 Plus NSAI In Luminal Advanced Breast Cancer |
| 312. | NCT04711252 | A Comparative Study of AZD9833 Plus Palbociclib Versus Anastrozole Plus Palbociclib in Patients With ER-Positive HER2 Negative Breast Cancer Who Have Not Received Any Systemic Treatment for Advanced Disease. |
| 313. | NCT04711824 | Study of Stereotactic Radiosurgery With Olaparib Followed by Durvalumab and Physician's Choice Systemic Therapy in Subjects With Breast Cancer Brain Metastases |
| 314. | NCT04707196 | A Study of Abemaciclib in Indian Women With Advanced Breast Cancer |
| 315. | NCT04705909 | Efficacy of Statin Addition to Neoadjuvant Chemotherapy Protocols for Breast Cancer |
| 316. | NCT04699630 | A Study of U3-1402 in Subjects With Metastatic Breast Cancer |
| 317. | NCT04690855 | A Study to Evaluate TAlazoparib, Radiotherapy and Atezolizumab in gBRCA 1/2 Negative Patients With PD-L1+ Metastatic Triple Negative Breast Cancer |
| 318. | NCT04683679 | A Study of Radiation Therapy With Pembrolizumab and Olaparib in Women Who Have Triple-Negative Breast Cancer |
| 319. | NCT04680442 | Safety of Continuing HER-2 Directed Therapy in Overt Left Ventricular Dysfunction |
| 320. | NCT04681287 | Exploratory Study of Advanced Breast Cancer in HER2 Positive Patients With Failure of Multi-line Therapy Treated by Combination of Inetetamab (Cipterbin) + PD-1 Inhibitor Combined With Utidelone. |
| 321. | NCT04681911 | Inetetamab Combined With Pyrotinib and Chemotherapy in the Treatment of HER2 Positive Metastatic Breast Cancer |
| 322. | NCT04676516 | A Phase II Window of Opportunity Trial of PRMT5 Inhibitor, GSK3326595, in Early Stage Breast Cancer |
| 323. | NCT04676997 | Neoadjuvant Study of Camrelizumab Plus Chemotherapy in Triple Negative Breast Cancer (TNBC) |
| 324. | NCT04677816 | Impact of Vitamin D Supplementation on the Rate of Pathologic Complete Response in Vitamin D Deficient Patients |
| 325. | NCT04675827 | De-escalation Adjuvant Chemo in HER2+/ER-/Node-neg Early BC Patients Who Achieved pCR After Neoadjuvant Chemo & Dual HER2 Blockade |
| 326. | NCT04669587 | ER+/HER2- Locally Advanced or Metastatic Breast Cancer (ENZENO Study) |
| 327. | NCT04673448 | Niraparib and TSR-042 for the Treatment of BRCA-Mutated Unresectable or Metastatic Breast, Pancreas, Ovary, Fallopian Tube, or Primary Peritoneal Cancer |
| 328. | NCT04665986 | Neoadjuvant Study of Navelbine in Patients With HER2 Positive Breast Cancer |
| 329. | NCT04666961 | Impact of Neoadjuvant Hormonal Therapy on the Surgical Management of Extensive Ductal Carcinomas in Situ |
| 330. | NCT04664829 | The Role of Bexarotene in Inducing Susceptibility to Chemotherapy in Metastatic TNBC |
| 331. | NCT04659499 | Nab-paclitaxel in Combination With Pyrotinib in Postoperative Adjuvant Therapy for HER2-positive Breast Cancer |
| 332. | NCT04650581 | Fulvestrant and Ipatasertib for Advanced HER-2 Negative and Estrogen Receptor Positive (ER+) Breast Cancer Following Progression on First Line CDK 4/6 Inhibitor and Aromatase Inhibitor |
| 333. | NCT04647916 | Testing Sacituzumab Govitecan Therapy in Patients With HER2-Negative Breast Cancer and Brain Metastases |
| 334. | NCT04646759 | Fulvestrant or Capecitabine Combined With Pyrotinib in HR+/HER2+ Metastatic Breast Cancer |
| 335. | NCT04639986 | Asian Study of Sacituzumab Govitecan (IMMU-132) in HR+/HER2- MBC |
| 336. | NCT04639271 | Pyrotinib, Trastuzumab And Abraxane in HER2-positive MBC With Brain Metastasis |
| 337. | NCT04634747 | Phase 2 Study to Evaluate PVX-410 + Pembrolizumab + Chemotherapy for Metastatic, PD-L1+ Triple-Negative Breast Cancer |
| 338. | NCT04631835 | Phase I Study of the HS-10352 in Patients With Advanced Breast Cancer |
| 339. | NCT04629846 | Study in Participants With Early-Stage or Locally Advanced Human Epidermal Growth Factor Receptor (HER) 2-Positive and Estrogen Receptor/Progesterone Receptor Negative Breast Cancer to Evaluate the Efficiency and Safety of Treatment With Trastuzumab Plus (+) QL1209/Pertuzumab + Docetaxel. |
| 340. | NCT04624711 | Eribulin Combined With Anlotinib in Metastatic HER2 Negative Breast Cancer |
| 341. | NCT04622319 | A Study of Trastuzumab Deruxtecan (T-DXd) Versus Trastuzumab Emtansine (T-DM1) in High-risk HER2-positive Participants With Residual Invasive Breast Cancer Following Neoadjuvant Therapy (DESTINY-Breast05) |
| 342. | NCT04613674 | A Study of Camrelizumab Plus Chemotherapy vs Placebo Plus Chemotherapy as Neoadjuvant Therapy in Participants With Triple Negative Breast Cancer (TNBC) |
| 343. | NCT04605575 | Pyrotinib Plus Vinorelbine in Participants With HER2-positive Previously Treated Locally Advanced or Metastatic Breast Cancer |
| 344. | NCT04602117 | ISPY-P1.01:Evaluating the Safety of Weekly Paclitaxel With Trastuzumab Duocarmazine (SYD985) in Patients With Metastatic Cancer |
| 345. | NCT04603183 | ABemaciclib, ET ± paclItaxel in aGgressive HR+/HER2- MBC trIaL |
| 346. | NCT04601116 | The MASTER Study (MAmmary Cancer STatin ER Positive Study) |
| 347. | NCT04596150 | Study to Evaluate the Safety and Antitumor Activity of CX-2009 Monotherapy and in Combination With CX-072 in Advanced Breast Cancer |
| 348. | NCT04595565 | Sacituzumab Govitecan in Primary HER2-negative Breast Cancer |
| 349. | NCT04588298 | A Study to Investigate the Biological Effects of AZD9833 in Women With ER-positive, HER2 Negative Primary Breast Cancer |
| 350. | NCT04584853 | PreOperative Endocrine Therapy for Individualised Care With Abemaciclib |
| 351. | NCT04584112 | A Study of the Safety, Efficacy, and Pharmacokinetics of Tiragolumab in Combination With Atezolizumab and Chemotherapy in Participants With Triple-Negative Breast Cancer |
| 352. | NCT04584255 | Niraparib + Dostarlimab In BRCA Mutated Breast Cancer |
| 353. | NCT04577963 | A Study of Fruquintinib in Combination With Tislelizumab in Advanced Triple Negative Breast Cancer |
| 354. | NCT04578106 | Omission of Surgery in Clinically Low-risk HER2positive Breast Cancer With High HER2 Addiction and a Complete Response Following Standard Anti-HER2-based Neoadjuvant Therapy |
| 355. | NCT04576143 | Efficacy and Safety of Dose-dense Chemotherapy (ddEC-ddP) for Neoadjuvant Chemotherapy of HER2-negative Breast Cancer |
| 356. | NCT04576455 | A Study Evaluating the Efficacy and Safety of Giredestrant Compared With Physician's Choice of Endocrine Monotherapy in Participants With Previously Treated Estrogen Receptor-Positive, HER2-Negative Locally Advanced or Metastatic Breast Cancer (acelERA Breast Cancer) |
| 357. | NCT04571437 | Letrozole and Metronomic Capecitabine in ER-positive HER2 Negative Advanced Breast Cancer (B-001 Study) |
| 358. | NCT04572295 | A Study of E7090 as Monotherapy and in Combination With Other Anticancer Agents in Participants With Estrogen Receptor Positive (ER+) and Human Epidermal Growth Receptor 2 Negative (HER2-) Recurrent/Metastatic Breast Cancer |
| 359. | NCT04569747 | A Single Arm Phase II Study of ADjuvant Endocrine Therapy, Pertuzumab, and Trastuzumab for Patients With Anatomic Stage I Hormone Receptor-positive, HER2-positive Breast Cancer |
| 360. | NCT04570956 | Oral Tamoxifen vs. TamGel vs. Control in Women With Atypical Hyperplasia or Lobular Carcinoma In Situ |
| 361. | NCT04568616 | Neoadjuvant ArOMatase Inhibitor Therapy for ER+ Breast Cancer (NAOMI) |
| 362. | NCT04568902 | Study of H3B-6545 in Japanese Women With Estrogen Receptor (ER)-Positive, Human Epidermal Growth Factor Receptor 2 (HER2)-Negative Breast Cancer |
| 363. | NCT04567420 | DNA-Guided Second Line Adjuvant Therapy For High Residual Risk, Stage II-III, Hormone Receptor Positive, HER2 Negative Breast Cancer |
| 364. | NCT04565054 | Adj. Dyn. Marker-adjusted Personalized Therapy Comparing Abemaciclib + SOC ET vs. SOC ET in Clinical or Genomic High Risk, HR+/HER2- EBC |
| 365. | NCT04563507 | Combined Immunotherapies in Metastatic ER+ Breast Cancer |
| 366. | NCT04556292 | A Phase II Study to Assess the Efficacy and Safety of SC10914 in the Metastatic Breast Cancer Patients |
| 367. | NCT04556773 | A Phase 1b Study of T-DXd Combinations in HER2-low Advanced or Metastatic Breast Cancer |
| 368. | NCT04553770 | Trastuzumab Deruxtecan Alone or in Combination With Anastrozole for the Treatment of Early Stage HER2 Low, Hormone Receptor Positive Breast Cancer |
| 369. | NCT04551495 | Neoadjuvant Study of Targeting ROS1 in Combination With Endocrine Therapy in Invasive Lobular Carcinoma of the Breast (ROSALINE) |
| 370. | NCT04547907 | Comparing the Efficacy of Nab-PHP and TCbHP in Neoadjuvant Therapy for HER2 Positive Operable Breast Cancer, A Multicenter, Randomized, Phase III Clinical Trial |
| 371. | NCT04546009 | A Study Evaluating the Efficacy and Safety of Giredestrant Combined With Palbociclib Compared With Letrozole Combined With Palbociclib in Participants With Estrogen Receptor-Positive, HER2-Negative Locally Advanced or Metastatic Breast Cancer (persevERA Breast Cancer) |
| 372. | NCT04544189 | Study Assessing the Efficacy and Safety of Treatment With Alpelisib Plus Fulvestrant Versus Placebo Plus Fulvestrant in Chinese Men and Postmenopausal Women With Advanced Breast Cancer |
| 373. | NCT04541433 | A Phase 1 Study of AZD9833 in Japanese Women With ER Positive, HER2 Negative Advanced Breast Cancer |
| 374. | NCT04539938 | A Study of Tucatinib Plus Trastuzumab Deruxtecan in HER2+ Breast Cancer |
| 375. | NCT04540692 | Evaluation of Sequencing of Anthracyclines and Taxanes for Locally Advanced HER2-negative Breast Cancer |
| 376. | NCT04538742 | A Phase 1b/2 Study of T-DXd Combinations in HER2-positive Metastatic Breast Cancer |
| 377. | NCT04537286 | First-line Nab-paclitaxel Plus Cisplatin Plus Carilizumab in mTNBC Patients |
| 378. | NCT04529044 | 177Lu-DOTATATE for the Treatment of Stage IV or Recurrent Breast Cancer |
| 379. | NCT04523857 | ABemacicliB or Abemaciclib and HydroxYchloroquine to Target Minimal Residual Disease in Breast Cancer |
| 380. | NCT04524000 | Study Assessing the Efficacy and Safety of Treatment With Alpelisib Plus Fulvestrant in Japanese Men and Postmenopausal Women With Advanced Breast Cancer |
| 381. | NCT04517292 | Eribulin Plus Cisplatin Versus Gemcitabine Plus Cisplatin in Triple Negative Breast Cancer (TNBC) |
| 382. | NCT04514419 | Treatment of Breast Cancer With Trastuzumab + HS627/ Pertuzumab + Docetaxel |
| 383. | NCT04514159 | A Study of ZN-c5 and Abemaciclib in Participants With Breast Cancer |
| 384. | NCT04509596 | DZD1516 in Combination With Trastuzumab and Capecitabine, or in Combination With T-DM1, in Patients With Metastatic HER2 Positive Breast Cancer |
| 385. | NCT04508803 | Combination of HX008 And Niraparib in GErm-line-mutAted Metastatic Breast Cancer |
| 386. | NCT04505826 | A Dose Escalation/Expansion Study of Oral OP-1250 in Subjects With Advanced and/or Metastatic HR+, HER2- Breast Cancer |
| 387. | NCT04504331 | Study of Infigratinib in Combination With Tamoxifen or With Fulvestrant and Palbociclib in Hormone Receptor Positive, HER2 Negative, FGFR Altered Advanced Breast Cancer |
| 388. | NCT04501523 | A Prospective, Phase II Trial Using ctDNA to Initiate Post-operation Boost Therapy After NAC in TNBC |
| 389. | NCT04502680 | Maintenance Treatment With Eribulin Mesylate Versus Observation in Triple Negative Breast Cancer Patients |
| 390. | NCT04498793 | Study of Tislelizumab Plus Chemotherapy vs Chemotherapy as Perioperative Treatment in Participants With HER2 Negative Breast Cancer |
| 391. | NCT04494425 | Study of Trastuzumab Deruxtecan (T-DXd) vs Investigator's Choice Chemotherapy in HER2-low, Hormone Receptor Positive, Metastatic Breast Cancer |
| 392. | NCT04494958 | Palbociclib and Binimetinib in Advanced Triple Negative Breast Cancer |
| 393. | NCT04489173 | TAS102 in Patients With ER-positive, HER2-negative Advanced Breast Cancer |
| 394. | NCT04489940 | Bintrafusp Alfa in High Mobility Group AT-Hook 2 (HMGA2) Expressing Triple Negative Breast Cancer |
| 395. | NCT04486911 | Pyrotinib Maleate, CDK4/6 Inhibitor and Letrozole in Combination for Stage II-III TPBC: a Phase II Trial |
| 396. | NCT04481113 | Abemaciclib and Niraparib Before Surgery for the Treatment of Hormone Receptor Positive HER2 Negative Breast Cancer |
| 397. | NCT04481763 | A Study of the Efficacy and Safety of Camrelizumab Plus Radiotherapy for Patients With Early Triple-Negative Breast |
| 398. | NCT04481932 | Trastuzumab Combined With Pyrrolidine and Chemotherapy for Locally HER2 Positive Breast Cancer |
| 399. | NCT04478266 | Amcenestrant (SAR439859) Plus Palbociclib as First Line Therapy for Patients With ER (+) HER2(-) Advanced Breast Cancer |
| 400. | NCT04468061 | Sacituzumab Govitecan +/- Pembrolizumab in Metastatic TNBC |
| 401. | NCT04469127 | A Phase I/II Multicenter, Open-Label Study of Lu-177-αvβ3-IAC, for the Treatment of Angiogenic Breast Cancer Patients. |
| 402. | NCT04464174 | Ipatasertib Plus Non-Taxane Chemotherapy for Advanced or Metastatic Triple-Negative Breast Cancer |
| 403. | NCT04465097 | Neoadjuvant Tucidinostat and Exemestane in Early Breast Cancer |
| 404. | NCT04461600 | A Study of AL101 Monotherapy in Patients With Notch Activated Triple Negative Breast Cancer |
| 405. | NCT04457596 | T-DM1 and Tucatinib Compared With T-DM1 Alone in Preventing Relapses in People With High Risk HER2-Positive Breast Cancer, the CompassHER2 RD Trial |
| 406. | NCT04460430 | Targeting EGFR/ERBB2 With Neratinib in Hormone Receptor (HR)-Positive/HER2-negative HER2-enriched Advanced/Metastatic Breast Cancer |
| 407. | NCT04454437 | Sacituzumab Govitecan in Chinese Patients With mTNBC of at Least 2 Prior Treatments |
| 408. | NCT04454528 | BreastVAX: Radiation Boost to Enhance Immune Checkpoint Blockade Therapy |
| 409. | NCT04452370 | Oral Etoposide Combined With Anlotinib in Advanced Triple Negative Breast Cancer |
| 410. | NCT04448886 | Sacituzumab Govitecan +/- Pembrolizumab In HR+ / HER2 - MBC |
| 411. | NCT04445844 | INCMGA00012 and Pelareorep for the Treatment of Metastatic Triple Negative Breast Cancer, IRENE Study |
| 412. | NCT04443348 | Pre-op Pembro + Radiation Therapy in Breast Cancer (P-RAD) |
| 413. | NCT04436744 | A Study Evaluating the Efficacy, Safety, and Pharmacokinetics of Giredestrant Plus Palbociclib Compared With Anastrozole Plus Palbociclib for Postmenopausal Women With Estrogen Receptor-Positive and HER2-Negative Untreated Early Breast Cancer (coopERA Breast Cancer) |
| 414. | NCT04437160 | Adjuvant Chemotherapy for Triple Negative Breast Cancer Patients With Residual Disease After Neoadjuvant Chemotherapy |
| 415. | NCT04434560 | Neoadjuvant Immunotherapy in Brain Metastases |
| 416. | NCT04432454 | Evaluation of Lasofoxifene Combined With Abemaciclib in Advanced or Metastatic ER+/HER2- Breast Cancer With an ESR1 Mutation |
| 417. | NCT04434040 | Atezolizumab + Sacituzumab Govitecan to Prevent Recurrence in TNBC (ASPRIA) |
| 418. | NCT04430595 | Multi-4SCAR-T Therapy Targeting Breast Cancer |
| 419. | NCT04425018 | MARGetuximab Or Trastuzumab (MARGOT) |
| 420. | NCT04427293 | Preoperative Lenvatinib Plus Pembrolizumab in Early-Stage Triple-Negative Breast Cancer (TNBC) |
| 421. | NCT04420598 | DS-8201a for trEatment of aBc, BRain Mets, And Her2[+] Disease |
| 422. | NCT04419181 | Feasibility of Chemotherapy De-escalation in Early-Stage HER2 Positive Breast Cancer |
| 423. | NCT04408118 | First Line Atezolizumab, Paclitaxel, and Bevacizumab (Avastin®) in mTNBC |
| 424. | NCT04403529 | Evaluating the Clinical Value of Traditional Chinese Medicine in the Adjuvant Therapy of Triple-negative Breast Cancer |
| 425. | NCT04398914 | Pyrotinib, Trastuzumab, Pertuzumab and Nab-paclitaxel as Neoadjuvant Therapy in HER2-positive Breast Cancer |
| 426. | NCT04400695 | A Study of RC48-ADC for the Treatment of Locally Advanced or Metastatic Breast Cancer With Low Expression of HER2 |
| 427. | NCT04398108 | A Study to Evaluate the Pharmacokinetics of Margetuximab in Chinese Patients With HER2+ MBC |
| 428. | NCT04395989 | An Umbrella Trial Based on Molecular Pathway for Patients With Metastatic TNBC. |
| 429. | NCT04389073 | Safety and Efficacy of Toripalimab in HER2- Metastatic Breast Cancer Patients Treated With Metronomic Vinorelbine |
| 430. | NCT04387630 | Neoadjuvant Chemotherapy With or Without Metformin in Early Breast Cancer. |
| 431. | NCT04383275 | Stage I HER2 Positive Invasive Breast Cancer De-escalation Study(IRIS) |
| 432. | NCT04373031 | Pembrolizumab, IRX-2, and Chemotherapy in Triple Negative Breast Cancer |
| 433. | NCT04367090 | Pharmacokinetic Study of Pyrotinib and Docetaxel in Combination With Trastuzumab in Patients With HER2 Metastatic Breast Cancer |
| 434. | NCT04355858 | Molecularly Targeted Umbrella Study in Luminal Advanced Breast Cancer |
| 435. | NCT04352777 | Impact of Endocrine Therapy and Abemaciclib on Host and Tumor Immune Cell Repertoire/Function in Advanced ER+/HER2- Breast Cancer |
| 436. | NCT04348747 | Dendritic Cell Vaccines Against Her2/Her3 and Pembrolizumab for the Treatment of Brain Metastasis From Triple Negative Breast Cancer or HER2+ Breast Cancer |
| 437. | NCT04337658 | Anti-HER2 Therapy + Fulvestrant/Capecitabine in Women With HR+, HER2+, Non-visceral Metastases Stage IV Breast Cancer |
| 438. | NCT04334330 | Palbociclib, Trastuzumab,Pyrotinib and Fulvestrant Treatment in Patients With Brain Metastasis From ER/PR Positive, HER-2 Positive Breast Cancer: A Multi-center, Prospective Study in China |
| 439. | NCT04335006 | A Phase 3 Study Comparing Carelizumab Plus Nab-paclitaxel and Apatinib, Carelizumab Plus Nab-paclitaxel, and Nab-paclitaxel in Patients With Advanced Triple Negative Breast Cancer. |
| 440. | NCT04333706 | A Dose Finding Phase 1 of Sarilumab Plus Capecitabine in HER2/Neu-Negative Metastatic Breast Cancer and a Single-arm, Historically-controlled Phase 2 Study of Sarilumab Plus Capecitabine in Stage I-III Triple Negative Breast Cancer With High-Risk Residual Disease (EMPOWER) |
| 441. | NCT04331067 | Cabiralizumab in Combination With Nivolumab and Neoadjuvant Chemotherapy in Patients With Localized Triple-negative Breast Cancer |
| 442. | NCT04329065 | Concurrent WOKVAC Vaccination, Chemotherapy, and HER2-Targeted Monoclonal Antibody Therapy Before Surgery for the Treatment of Patients With Breast Cancer |
| 443. | NCT04316169 | Hydroxychloroquine, Abemaciclib and Endocrine Therapy in Hormone Receptor Positive (HR+)/Her 2 Negative Breast Cancer |
| 444. | NCT04307329 | Monalizumab and Trastuzumab In Metastatic HER2-pOSitive breAst Cancer: MIMOSA-trial |
| 445. | NCT04305236 | Neo-Adjuvant Abemaciclib With Fulvestrant in Patients With ER/PR +HER Negative Breast Cancer |
| 446. | NCT04305496 | Capivasertib+Fulvestrant vs Placebo+Fulvestrant as Treatment for Locally Advanced (Inoperable) or Metastatic HR+/HER2- Breast Cancer |
| 447. | NCT04305834 | Abemaciclib and Endocrine Therapy in Older Patients With Breast Cancer. |
| 448. | NCT04303741 | A Phase II Trial of Camrelizumab in Combination With Apatinib and Eribulin in Patients With Advanced TNBC |
| 449. | NCT04301739 | to Evaluate Efficacy and Safety of HLX10 in Combination With Chemotherapy Versus Placebo in Combination With Chemotherapy as Neoadjuvant Therapy and HLX10 Versus Placebo as Adjuvant Therapy in Patients With Triple Negative Breast Cancer (TNBC) |
| 450. | NCT04300790 | Study to Evaluate the Effect of Metformin in the Prevention of HG in HR[+]/HER2[-] PIK3CA-mut Advanced BC Patients |
| 451. | NCT04298918 | A Study Evaluating the Safety and Efficacy of Venetoclax in Combination With Trastuzumab Emtansine in Patients With Previously Treated HER2-Positive Locally Advanced or Metastatic Breast Cancer |
| 452. | NCT04296370 | A Study of Fluzoparib±Apatinib Versus Chemotherapy Treatment of Physician's Choice in HER2-negative Metastatic Breast Cancer Patients With Germline BRCA Mutation |
| 453. | NCT04296942 | BN-Brachyury, Entinostat, Adotrastuzumab Emtansine and M7824 in Advanced Stage Breast Cancer (BrEAsT) |
| 454. | NCT04294225 | Anastrozole and Letrozole After Surgery for the Treatment of Stage I-III Breast Cancer |
| 455. | NCT04293276 | Study to Evaluate the Efficacy and Safety of SHR6390 Combined With Pyrotinib in HER2+ Advanced Breast Cancer |
| 456. | NCT04293393 | Neoadjuvant Study Chemotherapy vs Letrozole + Abemaciclib in HR+/HER2- High/Intermediate Risk Breast Cancer Patients |
| 457. | NCT04290793 | Neoadjuvant Chemotherapy With Pyrotinib, Epirubicin and Cyclophosphamide Followed by Taxanes and Trastuzumab for HER-2+ Breast Cancer |
| 458. | NCT04288089 | A Study of H3B-6545 in Combination With Palbociclib in Women With Advanced or Metastatic Estrogen Receptor-Positive Human Epidermal Growth Factor Receptor-2 (HER2)-Negative Breast Cancer |
| 459. | NCT04274933 | A Study to Evaluate the Safety and Tolerability of Venetoclax Tablets in Combination With Capecitabine Tablets in Adult Participants With Hormone Receptor-Positive, HER2-Negative Locally Advanced or Metastatic Breast Cancer Who Had Disease Progression During or After CDK4/6 Inhibitor Therapy |
| 460. | NCT04272801 | Neoadjuvant Endocrine Therapy Tolerance in Geriatric Early Stage ER+ Breast Cancer |
| 461. | NCT04266249 | CompassHER2-pCR: Decreasing Chemotherapy for Breast Cancer Patients After Pre-surgery Chemo and Targeted Therapy |
| 462. | NCT04265274 | Vinorelbine, Cisplatin, Disulfiram and Copper in CTC_EMT Positive Refractory Metastatic Breast Cancer. |
| 463. | NCT04261218 | Safety, Pharmacodynamics, Pharmacokinetics, and Efficacy of Tomivosertib Combined With Paclitaxel in Advanced Breast Cancer |
| 464. | NCT04251533 | Study Assessing the Efficacy and Safety of Alpelisib + Nab-paclitaxel in Subjects With Advanced TNBC Who Carry Either a PIK3CA Mutation or Have PTEN Loss |
| 465. | NCT04252768 | A Study in Hormone Receptor-positive Metastatic Breast Carcinoma Patients to Test a New Schedule of Efti (IMP321, Eftilagimod Alpha) as Adjunctive to a Weekly Treatment Regimen of Paclitaxel |
| 466. | NCT04253561 | Ipatasertib + Pertuzumab +Trastuzumab in Advanced HER2+ PI3KCA-mutant Breast Cancer Patients |
| 467. | NCT04255056 | Pyrotinib in Breast Cancer Patients With Poor Response to the Neoadjuvant Treatment of Trastuzumab and Pertuzumab |
| 468. | NCT04251169 | Pembrolizumab + Paclitaxel in Hormone Receptor-positive (HR+)/Human Epidermal Growth Factor Receptor 2-negative (HER2-) Non-luminal (by PAM50) Advanced Breast Cancer After Cyclin-dependent Kinase 4/6 (CDK4/6) Inhibitors Progression |
| 469. | NCT04247633 | High Risk ER+HER2- T1-2N0-1 Early Breast Cancer With Palbociclib Plus Endocrine Therapy(HIPEx) |
| 470. | NCT04248998 | Calorie Restriction With or Without Metformin in Triple Negative Breast Cancer |
| 471. | NCT04246502 | Capecitabine Plus Pyrotinib Versus Capecitabine Plus Trastuzumab and Pertuzumab in the First-line Treatment of HER2-positive Metastatic Breast Cancer |
| 472. | NCT04243616 | Cemiplimab in High Risk or Locally Advanced Hormone Receptor Positive HER2 Negative or Triple-Negative Breast Cancer |
| 473. | NCT04240106 | Niraparib Plus Aromatase Inhibitors for Luminal-like(HER2-,ER+) and gBRCA or HDR+ Metastatic Breast Cancer (LUZERN) |
| 474. | NCT04236310 | A Study of Neoadjuvant SHR6390 in Combination With Anastrozole, Pyrotinib, and Trastuzumab in Patients With ER+/HER2+ Breast Cancer. |
| 475. | NCT04230109 | Sacituzumab Govitecan In TNBC |
| 476. | NCT04224272 | A Study of ZW25 (Zanidatamab) With Palbociclib Plus Fulvestrant in Patients With HER2+/HR+ Advanced Breast Cancer |
| 477. | NCT04227327 | Study Evaluating Abemaciclib + Aromatase Inhibitors in HR+, HER2- Advanced Breast Cancer Patients (HERMIONE-7) |
| 478. | NCT04214288 | A Comparative Study of AZD9833 Versus Fulvestrant in Women With Advanced ER-Positive HER2-Negative Breast Cancer |
| 479. | NCT04215003 | A Clinical Trial of Breast Cancer Neo-adjuvant Therapy Based on Molecular Pathway in FUSCC |
| 480. | NCT04215146 | A Study to Assess Overall Response Rate by Inducing an Inflammatory Phenotype in Metastatic BReast cAnCEr With the Oncolytic Reovirus PeLareorEp in CombinaTion With Anti-PD-L1 Avelumab and Paclitaxel - BRACELET-1 Study |
| 481. | NCT04213898 | SHR-1210 Combined With Albumin-bound Paclitaxel and Epirubicin Neoadjuvant for Triple Negative Breast Cancer |
| 482. | NCT04208178 | Study of Alpelisib (BYL719) in Combination With Trastuzumab and Pertuzumab as Maintenance Therapy in Patients With HER2-positive Advanced Breast Cancer With a PIK3CA Mutation |
| 483. | NCT04199520 | Compare the Efficacy of Surgery Combined With Systemic Therapy and Pure Systemic Therapy in Patients With Stage Ⅳ Breast Cancer |
| 484. | NCT04197687 | TPIV100 and Sargramostim for the Treatment of HER2 Positive, Stage II-III Breast Cancer in Patients With Residual Disease After Chemotherapy and Surgery |
| 485. | NCT04190056 | Pembrolizumab and Tamoxifen With or Without Vorinostat for the Treatment of Estrogen Receptor Positive Breast Cancer |
| 486. | NCT04191382 | Phase 2 Window Study of SAR439859 (Amcenestrant) Versus Letrozole in Post-menopausal Patients With ER+, HER2- Pre-operative Post-menopausal Primary Breast Cancer |
| 487. | NCT04191499 | A Study Evaluating the Efficacy and Safety of Inavolisib + Palbociclib + Fulvestrant vs Placebo + Palbociclib + Fulvestrant in Patients With PIK3CA-Mutant, Hormone Receptor-Positive, Her2-Negative, Locally Advanced or Metastatic Breast Cancer |
| 488. | NCT04188119 | A Proof of Concept Window Trial of the IMmunological Effects of AveLumab and Aspirin in Triple-Negative Breast Cancer |
| 489. | NCT04176757 | A Study of ZN-c5 in Participants With Breast Cancer |
| 490. | NCT04158856 | Adjuvant Dual Anti-HER2 Therapy in Patients With Small, Node-negative, HER2-positive Breast Cancer |
| 491. | NCT04158947 | A Study of HER2+ Breast Cancer Patients With Active Brain Metastases Treated With Afatinib & T-DM1 vs. T-DM1 Alone |
| 492. | NCT04159818 | Immune Induction Strategies to Improve Response to Immune Checkpoint Blockade in Triple Negative Breast Cancer (TNBC) Patients |
| 493. | NCT04158362 | Endocrine Therapy With Abemaciclib or Chemotherapy as Initial Metastatic Treatment in ER+/HER2- Breast Cancer |
| 494. | NCT04142060 | Targeting the PAM50 Her2-Enriched Phenotype With Enzalutamide in Hormone Receptor-Positive/Her2-Negative Metastatic BC |
| 495. | NCT04139993 | RBX7455 Before Surgery for the Treatment of Operable Breast Cancer |
| 496. | NCT04137640 | Palbociclib + Letrozole Versus Epirubicin + Cyclophosphamide and Sequential Docetaxel as Neoadjuvant Chemotherapy |
| 497. | NCT04137653 | Treatment of Triple-negative Breast Cancer With Albumin-bound Paclitaxel as Neoadjuvant Therapy: a Prospective RCT |
| 498. | NCT04136782 | Albumin-bound Paclitaxel and Carboplatin Versus Epirubicin and Docetaxel for Triple-negative Breast Cancer |
| 499. | NCT04134598 | ExclUsive endocRine Therapy Or Partial Breast Irradiation for Women Aged ≥70 Years Early Stage Breast Cancer |
| 500. | NCT04134884 | Study of ASTX727 Plus Talazoparib in Patients With Triple Negative or Hormone Resistant/HER2-negative Metastatic Breast Cancer |
| 501. | NCT04132817 | A Study of Multiple Immune and Disease Treatment Combinations in Participants With ER+HER2- Breast Cancer That Has Spread |
| 502. | NCT04123704 | Sitravatinib in Metastatic Breast Cancer |
| 503. | NCT04120246 | Alpha-TEA and Trastuzumab for the Treatment of Refractory HER2+ Metastatic Breast Cancer |
| 504. | NCT04097756 | A Phase I Study of LX-039 Tablets |
| 505. | NCT04090567 | Olaparib With Cediranib or AZD6738 for the Treatment of Advanced or Metastatic Germline BRCA Mutated Breast Cancer |
| 506. | NCT04024462 | A Two-Arm Study to Evaluate the Pharmacokinetics, Efficacy, and Safety of Subcutaneous Administration of the Fixed-Dose Combination of Pertuzumab and Trastuzumab in Combination With Chemotherapy in Chinese Participants With HER2-Positive Early Breast Cancer |
| 507. | NCT04020575 | Autologous huMNC2-CAR44 T Cells for Breast Cancer Targeting Cleaved Form of MUC1 (MUC1*) |
| 508. | NCT03990896 | Evaluation of Talazoparib, a PARP Inhibitor, in Patients With Somatic BRCA Mutant Metastatic Breast Cancer: Genotyping Based Clinical Trial |
| 509. | NCT03989089 | Study of Pembrolizumab in Metastatic HER2-negative Breast Cancer Patients With APOBEC3B Mutation |
| 510. | NCT03988036 | A Study With Pembrolizumab in Combination With Dual Anti-HER2 Blockade With Trastuzumab and Pertuzumab in Early Breast Cancer Patients With Molecular HER2-enriched Intrinsic Subtype (Keyriched-1) |
| 511. | NCT03983395 | Study of ISB 1302 (CD3 Bispecific Ab) in HER2-positive Metastatic Breast Cancer |
| 512. | NCT03980509 | A "Window Trial" on Curcumin for Invasive Breast Cancer Primary Tumors |
| 513. | NCT03979508 | Abemaciclib in Treating Patients With Surgically Resectable, Chemotherapy Resistant, Triple Negative Breast Cancer |
| 514. | NCT03936933 | Pharmacodynamic Study Goserelin 3.6mg Injection Administered Subcutaneously in Premenopausal Patients With Breast Cancer |
| 515. | NCT03820141 | Durvalumab With Trastuzumab and Pertuzumab in HER2-Enriched Breast Cancer |
| 516. | NCT03804944 | Converting HR+ Breast Cancer Into an Individualized Vaccine |
| 517. | NCT03685331 | HOPE: Olaparib, Palbociclib and Fulvestrant in Patients With BRCA Mutation-associated, HR+, HER2-metastatic Breast Cancer |
| 518. | NCT03613181 | ANG1005 in Leptomeningeal Disease From Breast Cancer |
| 519. | NCT03554044 | T-VEC With Chemotherapy or Endocrine Therapy in Treating Participants With HER2- Negative Breast Cancer |
| 520. | NCT03492918 | Pembrolizumab in Combination With Paclitaxel in the Hormone Receptor-positive Metastatic Breast Cancer With High Tumor Mutational Burden Selected by Whole Exome Sequencing: Korean Cancer Study Group Trial (KCSG BR20-16) |
| 521. | NCT03324425 | Simvastatin Plus Dual Anti-HER2 Therapy for Metastatic Breast Cancer |
| 522. | NCT02802423 | Phase I/II, Evaluate the Safety and Efficacy of BLEX 404 With Docetaxel in Patients With Advanced/Metastatic Triple Negative Breast Cancer. |
