# Supplementary material for: Eligibility criteria in clinical trials in breast cancer: a cohort study
Source: BMC Med. 2023 Jul 3;21:240. doi: 10.1186/s12916-023-02947-y (PMC10318672; doi:10.1186/s12916-023-02947-y)
Supplement: Supplementary file 2 — Additional file 2: Table S1. Covariates affecting the odds of the presence of the upper age limits in clinical trials in breast cancer. [file 12916_2023_2947_MOESM2_ESM.docx]

**Table S1.** Covariates affecting the odds of the presence of the upper age limits in clinical trials in breast cancer

|  | **Univariate analysis** | |  | **Multivariate analysis** | |
| --- | --- | --- | --- | --- | --- |
|  | OR (95% CI) | *p* |  | Adjusted OR (95% CI) | *p* |
| **Breast cancer** |  |  |  |  |  |
| Early | Referent | - |  | Referent | - |
| Advanced | 0.69 (0.48-0.99) | 0.05 |  | 0.77 (0.46-1.30) | 0.33 |
| **Treatment** |  |  |  |  |  |
| C | Referent | - |  | Referent | - |
| C+T | 0.73 (0.33-1.59) | 0.43 |  | 0.64 (0.24-1.66) | 0.36 |
| T | 0.33 (0.15-0.74) | 0.01 |  | 0.53 (0.19-1.39) | 0.19 |
| H | 0.43 (0.16-1.09) | 0.078 |  | 1.04 (0.32-3.38) | 0.94 |
| H+T | 0.2 (0.09-0.44) | **<0.001** |  | 0.26 (0.1-0.66) | **0.006** |
| I | 0.13 (0.03-0.42) | **0.002** |  | 0.23 (0.04-1) | 0.06 |
| I+C | 0.52 (0.21-1.29) | 0.16 |  | 0.83 (0.26-2.61) | 0.74 |
| Other | 0.36 (0.16-0.77) | **0.01** |  | 0.59 (0.23-1.49) | 0.26 |
| **Phase** |  |  |  |  |  |
| 1 | Referent | - |  | Referent | - |
| 1/2 | 3.34 (1.73-6.99) | **0.001** |  | 2.24 (0.99-5.41) | 0.06 |
| 2 | 4.15 (1.99-9.26) | **<0.001** |  | 4.72 (1.8-13.13) | **0.002** |
| 2/3 | 2.64 (0.84-8.14) | 0.09 |  | 1.04 (0.24-4.27) | 0.95 |
| 3 | 3.09 (1.29-7.7) | **0.01** |  | 4.35 (1.49-13.35) | **0.008** |
| 4 | 16.36 (4.25-82.55) | **<0.001** |  | 12.35 (2.34-82.26) | **0.005** |
| **Sample size**^1^ |  |  |  |  |  |
|  | 0.99 (0.99-1) | 0.55 |  | 0.99 (0.99-1) | 0.52 |
| **Sponsor** |  |  |  |  |  |
| Industry | Referent | - |  | Referent | - |
| NIH | 0.27 (0.04-0.94) | 0.08 |  | 1.4 (0.2-6.19) | 0.69 |
| Other | 3.21 (2.22-4.67) | **<0.001** |  | 2.07 (1.25-3.43) | **0.005** |
| **Timeframe for primary endpoint assessment**^1^ | 0.99 (0.99-1) | 0.08 |  | 0.99 (0.99-0.99) | **0.03** |
|  |  |  |  |  |  |
| **Center location** |  |  |  |  |  |
| North America | Referent | - |  | Referent | - |
| Europe | 4.4 (2.35-8.63) | **<0.001** |  | 4.16 (2.03-9) | **<0.001** |
| Asia | 17.08 (9.44-32.79) | **<0.001** |  | 18.49 (9.13-40.06) | **<0.001** |
| Other | 5.67 (1.52-20.13) | **0.01** |  | 3.06 (0.66-13.25) | 0.13 |
| Intercontinental | 1.75 (0.73-4.05) | 0.2 |  | 2.51 (0.92-6.77) | 0.06 |

^1^ continuous variable. Abbreviations: C, chemotherapy; CI, confidence interval; H, hormonal therapy; I, immunotherapy; NIH, National Institutes of Health; OR, odds ratio; T, targeted therapy.
