# Supplementary material for: Eligibility criteria in clinical trials in breast cancer: a cohort study
Source: BMC Med. 2023 Jul 3;21:240. doi: 10.1186/s12916-023-02947-y (PMC10318672; doi:10.1186/s12916-023-02947-y)
Supplement: Supplementary file 3 — Additional file 3: Table S2. Covariates affecting the odds of the presence of strict exclusion criteria concerning comorbidities in clinical trials in breast cancer. [file 12916_2023_2947_MOESM3_ESM.docx]

**Table S2**. Covariates affecting the odds of the presence of strict exclusion criteria concerning comorbidities in clinical trials in breast cancer.

|  | **Univariate analysis** | |  | **Multivariate analysis** | |
| --- | --- | --- | --- | --- | --- |
|  | OR (95% CI) | *p* |  | Adjusted OR (95% CI) | *p* |
| **Breast cancer** |  |  |  |  |  |
| Early | Referent | - |  | Referent | - |
| Advanced | 0.68 (0.43-1.05) | 0.09 |  | 0.7 (0.4-1.2) | 0.2 |
| **Treatment** |  |  |  |  |  |
| C | Referent | - |  | Referent | - |
| C+T | 2.89 (1.08-7.8) | **0.03** |  | 2.45 (0.85-7.02) | 0.1 |
| T | 1.42 (0.56-3.45) | 0.45 |  | 1.31 (0.47-3.5) | 0.59 |
| H | 0.68 (0.24-1.86) | 0.45 |  | 0.78 (0.25-2.4) | 0.66 |
| H+T | 0.85 (0.36-1.92) | 0.71 |  | 0.91 (0.35-2.22) | 0.84 |
| I | 2.47 (0.66-12.08) | 0.21 |  | 2.25 (0.54-11.97) | 0.29 |
| I+T | 0.94 (0.34-2.59) | 0.91 |  | 0.79 (0.26-2.42) | 0.69 |
| Other | 1.48 (0.61-3.46) | 0.37 |  | 1.45 (0.55-3.64) | 0.43 |
| **Phase** |  |  |  |  |  |
| 1 | Referent | - |  | Referent | - |
| 1/2 | 3.19 (1.74-5.79) | **<0.001** |  | 2.76 (1.41-5.4) | **0.003** |
| 2 | 1.23 (0.63-2.38) | 0.54 |  | 1.51 (0.67-3.44) | 0.32 |
| 2/3 | 0.88 (0.32-2.52) | 0.81 |  | 0.62 (0.19-2) | 0.42 |
| 3 | 2.28 (0.96-5.77) | 0.07 |  | 2.18 (0.88-5.73) | 0.1 |
| 4 | 3.22 (0.78-21.95) | 0.15 |  | 3.04 (0.61-23.31) | 0.21 |
| **Sample size**^1^ |  |  |  |  |  |
|  | 0.99 (0.99-1) | 0.03 |  | 0.99 (0.99-1) | 0.22 |
| **Sponsor** |  |  |  |  |  |
| Industry | Referent | - |  | Referent | - |
| NIH | 1.04 (0.39-3.28) | 0.94 |  | 0.7 (0.22-2.51) | 0.56 |
| Other | 1.31 (0.86-2.02) | 0.22 |  | 0.82 (0.47-1.4) | 0.46 |
| **Timeframe for primary endpoint assessment**^1^ |  |  |  |  |  |
|  | 0.99 (0.99-1) | 0.34 |  | 1 (0.99-1) | 0.44 |
| **Center location** |  |  |  |  |  |
| North America | Referent | - |  | Referent | - |
| Europe | 1.3 (0.69-2.46) | 0.42 |  | 1.23 (0.61-2.49) | 0.56 |
| Asia | 0.95 (0.54-1.64) | 0.86 |  | 0.98 (0.51-1.86) | 0.96 |
| Other | 0.35 (0.1-1.27) | 0.1 |  | 0.26 (0.07-1.04) | **0.04** |
| Intercontinental | 0.34 (0.17-0.66) | **0.001** |  | 0.43 (0.19-0.94) | **0.03** |

^1^ continuous variable. Abbreviations: C, chemotherapy; CI, confidence interval; H, hormonal therapy; I, immunotherapy; NIH, National Institutes of Health; OR, odds ratio; T, targeted therapy.
