# Supplementary material for: Eligibility criteria in clinical trials in breast cancer: a cohort study
Source: BMC Med. 2023 Jul 3;21:240. doi: 10.1186/s12916-023-02947-y (PMC10318672; doi:10.1186/s12916-023-02947-y)
Supplement: Supplementary file 4 — Additional file 4: Table S3. Covariates affecting the odds of the presence of the exclusion criteria involving the performance status of the patient in clinical trials in breast cancer. [file 12916_2023_2947_MOESM4_ESM.docx]

**Table S3**. Covariates affecting the odds of the presence of the exclusion criteria involving the performance status of the patient in clinical trials in breast cancer.

|  | **Univariate analysis** | |  | **Multivariate analysis** | |
| --- | --- | --- | --- | --- | --- |
|  | OR (95% CI) | *p* |  | Adjusted OR (95% CI) | *p* |
| **Breast cancer** |  |  |  |  |  |
| Early | Referent | - |  | Referent | - |
| Advanced | 0.77 (0.52-1.15) | 0.21 |  | 0.45 (0.27-0.74) | **0.002** |
| **Treatment** |  |  |  |  |  |
| C | Referent | - |  | Referent | - |
| C+T | 3.43 (1.47-8.09) | **0.004** |  | 2.6 (1.05-6.46) | **0.03** |
| T | 1.84 (0.82-4.13) | 0.14 |  | 1.43 (0.58-3.52) | 0.43 |
| H | 0.76 (0.3-1.94) | 0.57 |  | 0.52 (0.18-1.5) | 0.23 |
| H+T | 1.83 (0.84-3.96) | 0.12 |  | 1.54 (0.66-3.57) | 0.32 |
| I | 1.43 (0.49-4.32) | 0.52 |  | 2.11 (0.62-7.53) | 0.24 |
| I+T | 2.54 (0.96-7.05) | 0.06 |  | 1.81 (0.62-5.47) | 0.28 |
| Other | 1.33 (0.62-2.84) | 0.46 |  | 1.57 (0.68-3.6) | 0.29 |
| **Phase** |  |  |  |  |  |
| 1 | Referent | - |  | Referent | - |
| 1/2 | 1.08 (0.59-1.91) | 0.81 |  | 0.81 (0.4-1.58) | 0.54 |
| 2 | 0.98 (0.49-1.93) | 0.95 |  | 0.53 (0.22-1.25) | 0.15 |
| 2/3 | 0.83 (0.29-2.48) | 0.72 |  | 0.53 (0.15-1.85) | 0.31 |
| 3 | 0.64 (0.29-1.43) | 0.28 |  | 0.55 (0.23-1.32) | 0.18 |
| 4 | 1 (0.29-4.04) | 1 |  | 0.66 (0.15-3.33) | 0.6 |
| **Sample size**^1^ |  |  |  |  |  |
|  | 1 (0.99-1) | 0.82 |  | 0.99 (0.99-1) | 0.19 |
| **Sponsor** |  |  |  |  |  |
| Industry | Referent | - |  | Referent | - |
| NIH | 0.19 (0.07-0.48) | **<0.001** |  | 0.34 (0.11-0.98) | 0.05 |
| Other | 0.52 (0.35-0.77) | **0.001** |  | 0.35 (0.21-0.57) | **<0.001** |
| **Timeframe for primary endpoint assessment**^1^ |  |  |  |  |  |
|  | 1 (0.99-1) | 0.99 |  | 1 (0.99-1) | 0.81 |
| **Center location** |  |  |  |  |  |
| North America | Referent | - |  | Referent | - |
| Europe | 1.99 (1.2 -3.32) | **0.008** |  | 2.16 (1.21-3.92) | **0.009** |
| Asia | 3.14 (1.94-5.15) | **<0.001** |  | 4.31 (2.4-7.91) | **<0.001** |
| Other | 0.62 (0.17-2.02) | 0.43 |  | 0.75 (0.2-2.71) | 0.66 |
| Intercontinental | 2.89 (1.48-5.91) | **0.002** |  | 3.46 (1.55-8.14) | **0.003** |

^1^ continuous variable. Abbreviations: C, chemotherapy; CI, confidence interval; H, hormonal therapy; I, immunotherapy; NIH, National Institutes of Health; OR, odds ratio; T, targeted therapy.
